# Identification and quantitation of clinically relevant microbes in patient samples: Comparison of three k-mer based classifiers for speed, accuracy, and sensitivity

**DOI:** 10.1371/journal.pcbi.1006863

**Published:** 2019-11-22

**Authors:** George S. Watts, James E. Thornton, Ken Youens-Clark, Alise J. Ponsero, Marvin J. Slepian, Emmanuel Menashi, Charles Hu, Wuquan Deng, David G. Armstrong, Spenser Reed, Lee D. Cranmer, Bonnie L. Hurwitz

**Affiliations:** 1 University of Arizona Cancer Center and Department of Pharmacology, University of Arizona, Tucson, Arizona, United States of America; 2 Department of Biosystems Engineering, University of Arizona, Tucson, Arizona, United States of America; 3 Department of Medicine, University of Arizona, Tucson, Arizona, United States of America; 4 Department of Biomedical Engineering, University of Arizona, Tucson, Arizona, United States of America; 5 Arizona Center for Accelerated Biomedical Innovation, University of Arizona, Tucson, Arizona, United States of America; 6 Honor Health Hospital, Scottsdale, Arizona, United States of America; 7 Dignity Health Chandler Regional Medical Center, Chandler, Arizona, United States of America; 8 Department of Endocrinology, Multidisciplinary Diabetic Foot Medical Center, Affiliated Central Hospital of Chongqing University, Chongqing, China; 9 Southwestern Academic Limb Salvage Alliance (SALSA), Department of Surgery, Keck School of Medicine of University of Southern California, Los Angeles, California, United States of America; 10 University of Arizona Department of Family and Community Medicine, Tucson, Arizona, United States of America; 11 Department of Medicine, University of Washington and Fred Hutchinson Cancer Research Center, and Seattle Cancer Care Alliance, Seattle, Washington, United States of America; 12 BIO5 Institute, University of Arizona, Tucson, Arizona, United States of America; DAL, CANADA

## Abstract

Infections are a serious health concern worldwide, particularly in vulnerable populations such as the immunocompromised, elderly, and young. Advances in metagenomic sequencing availability, speed, and decreased cost offer the opportunity to supplement or even replace culture-based identification of pathogens with DNA sequence-based diagnostics. Adopting metagenomic analysis for clinical use requires that all aspects of the workflow are optimized and tested, including data analysis and computational time and resources. We tested the accuracy, sensitivity, and resource requirements of three top metagenomic taxonomic classifiers that use fast k-mer based algorithms: Centrifuge, CLARK, and KrakenUniq. Binary mixtures of bacteria showed all three reliably identified organisms down to 1% relative abundance, while only the relative abundance estimates of Centrifuge and CLARK were accurate. All three classifiers identified the organisms present in their default databases from a mock bacterial community of 20 organisms, but only Centrifuge had no false positives. In addition, Centrifuge required far less computational resources and time for analysis. Centrifuge analysis of metagenomes obtained from samples of VAP, infected DFUs, and FN showed Centrifuge identified pathogenic bacteria and one virus that were corroborated by culture or a clinical PCR assay. Importantly, in both diabetic foot ulcer patients, metagenomic sequencing identified pathogens 4–6 weeks before culture. Finally, we show that Centrifuge results were minimally affected by elimination of time-consuming read quality control and host screening steps.

## Author summary

Currently, the gold standard for identifying pathogens that are causing infection is to attempt growth in culture followed by identification based on physical characteristics such as shape and metabolic profile. However, many organisms do not grow in culture or are overgrown by faster growing organisms that out-compete them. Another method to identify pathogens in infections is to sequence the DNA in the samples and use that DNA sequence to identify the pathogens present—a process called metagenomic sequencing. Analyzing clinical metagenomic data can be difficult given the amount of data generated, high levels of human DNA contamination and a lack of well-defined bioinformatics methods. In this study, three leading software tools were compared for identification and quantitation of microbes in metagenomic data. One tool, called Centrifuge, reliably identified microbes present at just 1% relative abundance while requiring less computer time and resources than the others to which it was compared. Moreover, we found that Centrifuge results changed minimally when time-consuming quality control and host-screening steps were eliminated. We also examined Centrifuge’s performance in real-word clinical data sets showing that Centrifuge identified the same pathogens as culture.

This is a *PLOS Computational Biology* Methods paper.

## Introduction

Intubated, diabetic, and neutropenic patients are susceptible to infections, yet current culture-based methods for identifying pathogens from clinical samples often fail [1–5]. Without diagnostic information, clinicians rely on empiric antibiotic therapy assuming that the organism is bacterial and susceptible to the selected antibiotic therapy. Metagenomic sequencing of clinical samples offers an approach that bypasses and overcomes many drawbacks of culture, however, mining the resulting metagenomic sequence can be slow and error-prone given the volume of reads, host read contamination, and lack of well-defined bioinformatics methods. We hypothesized that metagenomic sequencing and analysis with a recent k-mer based taxonomic classifier would provide rapid results while accurately matching both the known profiles of predefined samples and culture results from clinical samples. As such, the goal of our study was to assess three leading tools for taxonomic classification and quantitation of metagenomic data using clinically relevant datasets with an eye toward reducing computing time without sacrificing accuracy.

The current gold standard for clinical diagnosis of infections relies on isolating organisms by culture-based methods followed by microscopic and phenotypic identification combined with drug resistance testing. Methods for identifying pathogens that rely on culture have several drawbacks including fastidious bacteria, the time required for growth in culture, and the difficulty targeting viruses, fungi, and parasites. Because of these problems, culture has a high failure rate when rare, fastidious or non-bacterial organisms are responsible. For example, in a study of neutropenic patients the efficiency of culture-based diagnosis of blood stream infections was found to be low, with only ~16% (609 of 3,756) of febrile neutropenic patients found to be culture positive [4]. Compared to patients in whom no culture was taken, the hazard ratio of dying was nearly four-fold higher in culture-negative patients and higher still for culture-positive patients. The higher hazard ratio of dying in culture-negative patients suggests that many culture negative patients do indeed have an infection, and the high cost in lives when cultures fail.

In contrast to culture, identifying pathogens directly from biological samples via DNA sequencing can overcome many of the limitations of culture-based identification and may improve the number and speed of diagnoses [2,6]. In such an analysis, both the identity and relative abundance of the organisms present are important, as well as detecting the presence of drug resistance genes. While k-mer based tools such as MASH, Libra, and SIMKA are useful for comparing metagenomic content, they do not produce abundance estimates [7–9]. In contrast, read-by-read taxonomic classifiers such as Centrifuge, CLARK, and KrakenUniq can both identify and quantitate the organisms present in a metagenome. When applied to clinical samples, the rate of false positives and false negatives, as well as the speed and computational requirements for analysis are critical. For these reasons, direct analysis of pathogens using metagenomic methods, along with detection of drug resistance genes, has been referred to as the holy grail of molecular methods of infection diagnosis [10].

Presently, there are two metagenomic sequencing methods: marker gene sequencing and whole genome shotgun (WGS) sequencing [10,11]. Marker gene sequencing uses the 16S ribosomal RNA (16S rRNA) gene or other marker genes (e.g. the internal transcribed spacer region for fungi) to differentiate between bacteria or other targeted organisms based on variations in gene sequence. 16S rRNA sequencing and has been used extensively in microbiome surveys, as well as in the particular milieu of diabetic foot ulcer (DFU), ventilator acquired pneumonia (VAP), and bacteremia [12–16]. Because marker gene sequencing uses PCR to amplify the target gene sequences, it can detect organisms even when significant background is present, however, 16S rRNA sequencing has important drawbacks with respect to clinical applications. Drawbacks include “blind spots” in identification (e.g. *Escherichia coli* versus *Shigella flexneri*) due to sequence similarity between species and the inability to identify drug resistance (e.g. methicillin resistant *Staphylococcus aureus* versus methicillin-sensitive *S*. *Aureus*) [17,18]. In addition, detecting fungi requires different amplicons than bacteria, there are no universal primers for detecting viruses, and drug resistance detection requires specifically targeting the genes responsible with additional amplicons. Despite these limitations, 16S rRNA sequencing has been used to identify pathogens in bacteremia with increasing frequency [12,19]. By comparison, metagenomic data can yield fundamental insight into complex mixtures of microbial communities, their abundance and functional potential (including antibiotic resistance), and representation across all domains of life, including viruses. However, because metagenomics data can contain tens of millions of reads, efficient classification algorithms need to be employed to ensure reasonable runtime and computational requirements.

The potential of metagenomic WGS in clinical settings has been demonstrated in a broad range of infection scenarios including leptospirosis [20], nosocomial transmission of a drug-resistant bacteria [21], foodborne illness [22], infectious disease outbreaks [23], and recently in a prospective study of meningitis and encephalitis [24]. Despite successes using metagenomic shotgun sequencing to identify pathogens, routine application in clinical settings requires accurate, efficient classification, minimizing sample contamination, and rapid sample analysis [11,25–27]. For example, while a small group of studies have reported on high-throughput metagenomic sequencing for identifying pathogens from immunocompromised patients, the samples were not enriched for microbes, resulting in less than 1% of reads being pathogen-specific [28,29] dramatically reducing the diagnostic possibilities from the data [30]. To begin addressing these deficiencies, we developed a molecular approach to increase the proportion of pathogen-derived reads in samples and applied it to samples obtained from three groups of patients, those with: 1) ventilator acquired pneumonia; 2) infected diabetic foot ulcers that eventually resulted in amputation and 3) febrile neutropenia (FN) during anti-tumor therapy.

While there are no standards for analyzing metagenomic WGS data obtained from clinical samples, there have been recent innovations in taxonomic classification algorithms that make it possible to rapidly quantify microbial species directly from reads in metagenomic datasets. McIntyre *et al*. [31] evaluated the performance of three broad types of taxonomic classifiers based on alignment method, marker gene identification, and k-mer matches. Results showed that k-mer based algorithms, used in tools like CLARK [32] and Kraken [33], achieve high accuracy and reduced computational time compared to the other two classes of algorithms. These advantages make k-mer based classifiers particularly promising for the rapid identification and quantitation of pathogens in patient samples. Since the McIntyre study, new tools (Centrifuge [34]) and updates of previous tools (KrakenUniq and CLARK) were released. Centrifuge implemented memory-efficient indexing based on the Burrows-Wheeler transform [35,36] and a Ferragina-Manzini (FM) index [37] which allow storage of a large number of reference genome sequences without affecting Centrifuge’s memory requirements. KrakenUniq [38] builds on the previously released Kraken by using the cardinality estimation algorithm HyperLogLog for counting the number of unique *k*-mers identified for each taxon thus enabling confirmation that a detected taxon has even coverage across its genome. Lastly, CLARK was found by McIntyre *et al*. to have both higher accuracy and precision than other state of the art classifiers while taking advantage of multi-core architectures. While these innovations are promising for the detection of pathogens in clinical samples, to our knowledge, no study has directly compared these new k-mer based classifiers or investigated their accuracy with clinical samples.

Here we report the accuracy, sensitivity and computational requirements of three k-mer based taxonomic classification tools (CLARK, Centrifuge, and KrakenUniq) using a series of well-defined *in silico* and biological benchmark datasets [18,31,38]. We further report Centrifuge’s performance relative to culture in samples obtained from normal blood and patients. The clinical samples include (1) longitudinal ventilator exudate samples from two patients that developed VAP, (2) longitudinal curettage and tissue samples from two patients with infected DFUs that led to amputation, and (3) whole blood from three patients with FN. The clinical datasets were selected to show the spectrum of potential clinical infections ranging from: no infection (normal blood), infection with a single well-defined clinical microbe (VAP), polymicrobial infection (DFU), and infection with atypical pathogens (FN). Lastly, we tested the effect of quality control and host-screening on the classification of reads by Centrifuge. This work provides a foundation for analysis of metagenomic data from clinical samples using open-source software that requires minimal computational resources while providing rapid and accurate identification of pathogens. To promote reusability and further benchmarking, Centrifuge was added as a free web-based App in iMicrobe (http://iMicrobe.us, [39]) along with a benchmark dataset. In addition, singularity containers and source code for KrakenUniq (https://github.com/hurwitzlab/krakenuniq) and Centrifuge (https://github.com/hurwitzlab/centrifuge) were placed in GitHub under the MIT open source license.

## Results

### Accuracy of classification and abundance estimates in controlled mixtures of bacteria

**Fig 1:**
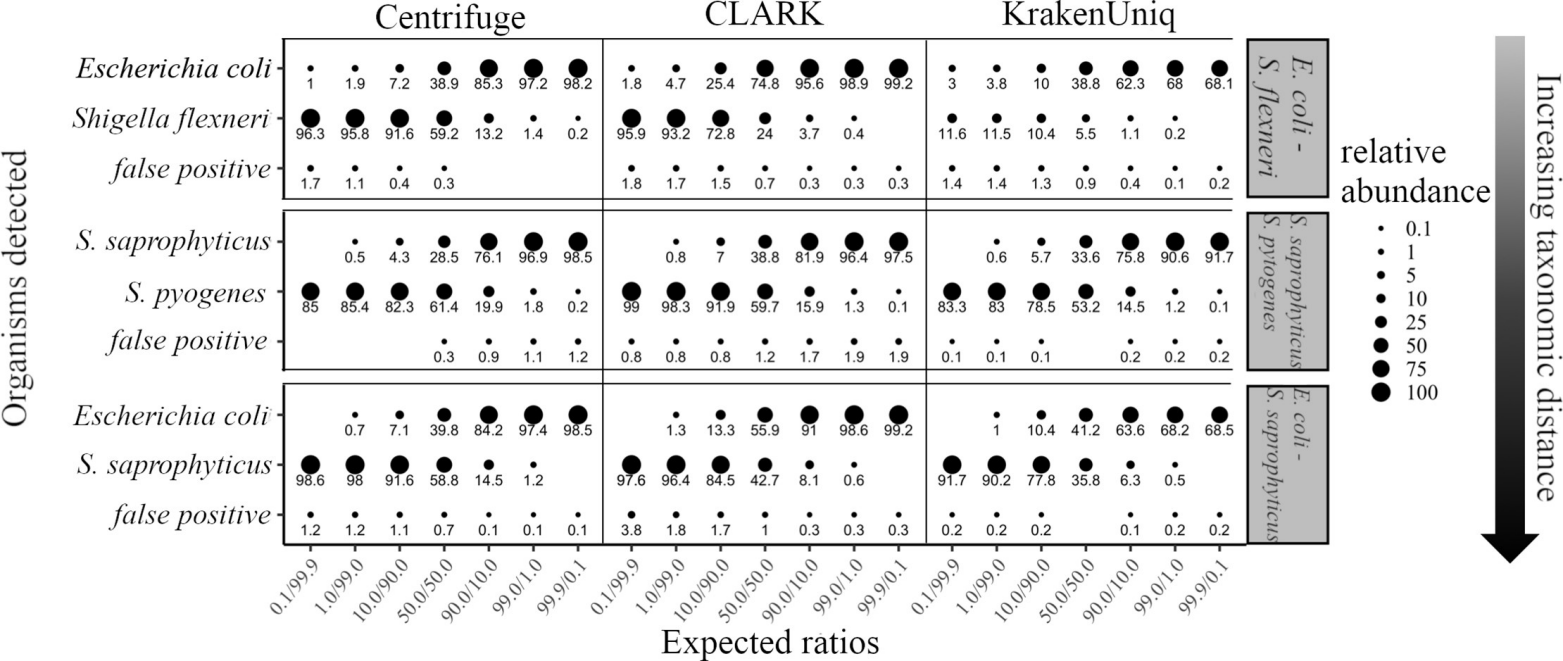
Accuracy of identification and abundance for three binary mixtures of bacteria using Centrifuge, CLARK, and KrakenUniq. After quality control, sequences were analyzed by Centrifuge, CLARK, and KrakenUniq using default settings and databases. Abundance estimates were filtered to include only organisms classified at the species or strain-level with at least 0.1% abundance. False positive abundance was calculated by summing the relative abundances of any organism identified by the classifiers that was not part of the mixture. The relative abundance of organisms identified by each tool is represented by circle size with actual values displayed below; values that are zero have no circle.

Because closely related, clinically important bacteria can have diametric clinical consequences, (e.g., *E*. *coli* is a normal commensal while *S*. *flexneri* causes dysentery), we used clinically relevant bacterial sequence datasets to test each classifier’s accuracy and threshold for detection. Classifiers were run using their default databases, settings, and abundance calculations. With respect to abundance calculations, Centrifuge normalizes relative abundance by genome size after calculating the percent of reads classified to each organism, while CLARK and KrakenUniq simply report relative abundance as percent of reads classified for each organism. Three sets of binary bacterial mixtures were selected to represent taxonomic distances from the species to phylum-level. The mixtures represent a three-log range of relative abundance with each organism ranging from 0.1% to 99.9% of the mixture (Fig 1). Centrifuge, CLARK, and KrakenUniq correctly identified all four species present in the mixtures and misidentified a maximum of four percent of the reads in any of the 18 combinations sequenced (false positives, Fig 1). Centrifuge was sensitive to the lowest relative abundance (0.1%) in three out of six opportunities, while CLARK and KrakenUniq identified the lowest relative abundance in two of six opportunities. CLARK and Centrifuge were equivalent in their accuracy of relative abundance, while KrakenUniq had particular difficulty estimating the relative abundances of *S*. *flexneri* and *E*. *coli* due to its strategy of assigning reads that match closely related organisms to the next highest shared taxonomic level. Reads matching phage present in the mixtures were classified and quantitated by Centrifuge, CLARK and KrakenUniq separately from their host genomes and were not included in the relative abundance estimates of their hosts, thus reducing the number of reads assigned to their hosts. Despite the effect of phage matches and false positive classifications, the coefficient of determination (R^2^) for the three mixtures quantitated by Centrifuge was 0.99 for *E*. *coli*/*S*. *flexneri*, 0.99 for *S*. *saprophyticus*/*S*. *pyogenes*, and 0.96 for *E*. *coli*/*S*. *saprophyticus*. Similarly, the coefficients of determination for CLARK were 0.99, 0.99, and 0.995 while those of KrakenUniq were 0.43, 0.99, and 0.96. Importantly, Centrifuge and CLARK were able to discriminate between organisms as difficult to separate as *E*. *coli* and *S*. *flexneri* while KrakenUniq could not.

### Accuracy of identification and abundance estimates in a staggered mock bacterial community

**Fig 2:**
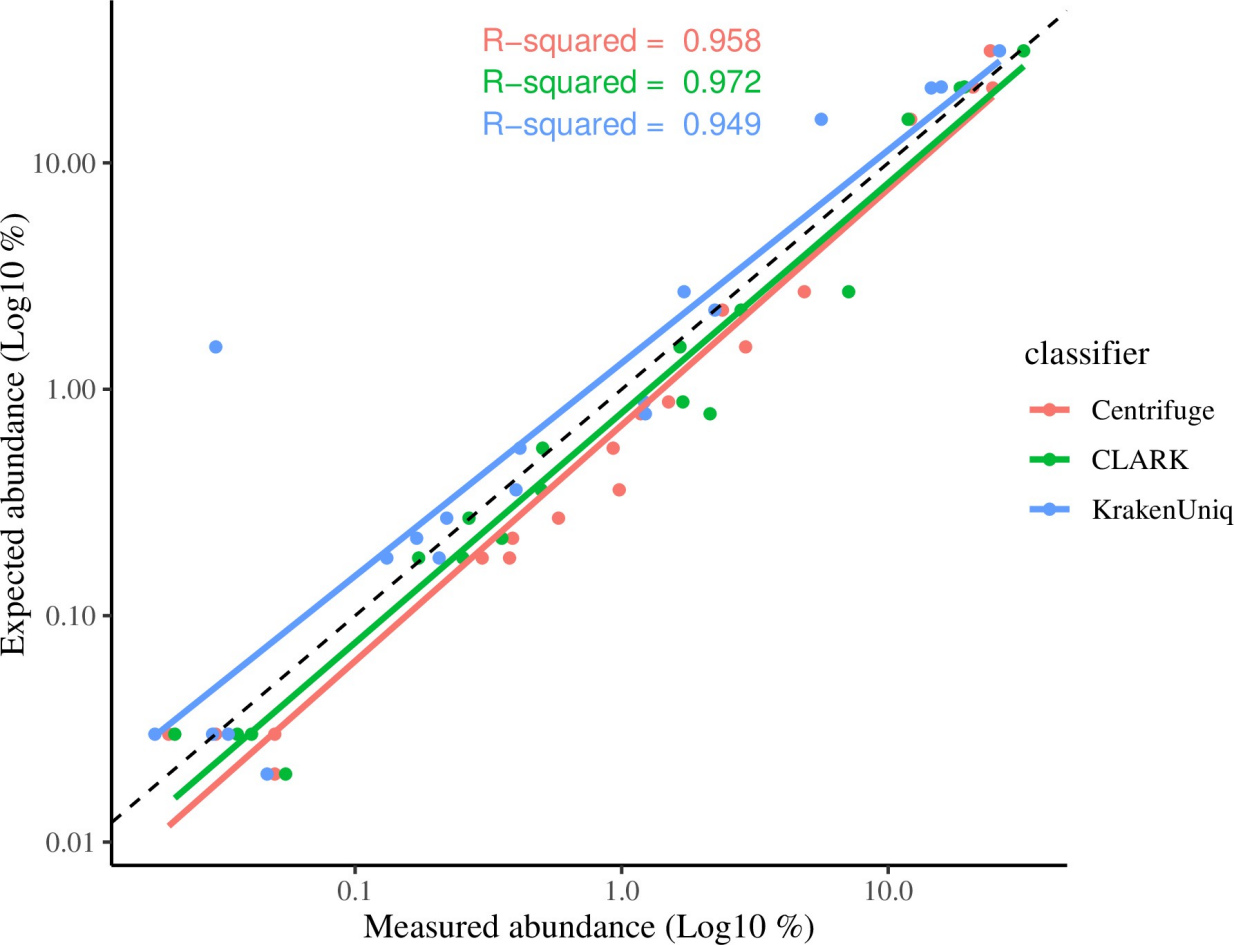
Centrifuge, CLARK, and KrakenUniq abundance estimates versus expected for a staggered abundance mock community. After quality control, the staggered mock community sequence was analyzed with Centrifuge, CLARK and KrakenUniq using default settings and databases. Abundance results were filtered to include only organisms classified at the species or strain-level with at least 0.1% abundance. The expected abundance of the organisms present in the community was plotted against the abundance reported by each classifier and the R^2^ calculated. The black dotted line represents perfect correlation with known relative abundances.

The three classifiers were next compared using a more complex mock community of 20 bacterium present in varying relative abundances. As the standard KrakenUniq and Centrifuge databases did not contain *Actinomyces odontolyticus* and therefore could not detect this organism in the mock community, *A*. *odontolyticus* was removed from the analysis. All three classifiers were able to detect the presence of the remaining 19 organisms, however, CLARK reported five false positives (two *Shigella sp*., two *Staphylococcus sp*. and *Corynebacterium pseudotuberculosis*) while KrakenUniq reported 9 false positives (three *Shigella sp*., two *Staphylococcus sp*., *Escherichia albertii*, *Salmonella enterica*, *Streptococcus troglodytae*, and *Klebsiella pneumoniae*). In contrast to CLARK and KrakenUniq, Centrifuge did not produce any false positives, and only reported additional hits to phages that infect the organisms present in the mock community. As with the binary bacterial mixtures, CLARK and Centrifuge had the most accurate relative abundance estimates, while KrakenUniq had a severe outlier (*Streptococcus agalactiae*) estimated as 1.54% when expected was 0.030%). The relative abundance of the 19 organisms in the mock community was graphed against their known abundance and R^2^ values calculated (Fig 2). The R^2^ values were nearly identical: 0.97 for CLARK, 0.96 for Centrifuge, and 0.95 for KrakenUniq. Overall, most estimated abundances made by Centrifuge and CLARK fell below the perfect fit represented by the dotted line in Fig 2, indicating they tended to overestimate relative abundance values, especially the lowest abundances. In contrast, KrakenUniq tended to underestimate the abundance values. Importantly, all three classifiers were able to detect the presence of all four organisms in the mock community with relative abundances of <0.03%.

### Further benchmarking of Centrifuge and KrakenUniq using 31 additional predefined datasets

Previously, McIntyre et al. [31] and Breitwieser et al. [38] reported on the performance of a broad variety of taxonomic classifiers using 31 known, predefined datasets (“truth sets”) composed of 21 *in silico*, and 10 biological, metagenomes obtained from several different sequencing platforms. To place Centrifuge within the context of this large dataset and the prior analyses in the literature, we analyzed the 31 “truth sets” with Centrifuge and KrakenUniq. CLARK was not included in these additional benchmarking analyses as the random access memory (RAM) required became prohibitive for analysis. Overall, Centrifuge and KrakenUniq had similar F1 and recall scores at the species level (Table 1).

**Table 1:** Average F1 and recall scores for Centrifuge and KrakenUniq at the species level for 21 *in silico* and 10 biological datasets.

	Biological datasets		*in silico* datasets	
	F1	recall	F1	recall
**KrakenUniq**	0.89	0.91	0.87	0.84
**Centrifuge**	0.82	0.92	0.90	0.89

### Comparison of the three classifiers based on computational resources used and runtime

When computational RAM and runtime were compared, there was a striking difference between Centrifuge and the other two classifiers. Relative to Centrifuge, CLARK and KrakenUniq required significantly more memory and time to analyze the staggered mock bacterial community (Table 2). Given Centrifuge’s performance in terms of accuracy of identification and quantitation, fewer false positives in the mock community, and lower memory and runtime, further investigation was limited to Centrifuge.

**Table 2:** Comparison of computational resources and runtime required by Centrifuge, CLARK and KrakenUniq to analyze the bacterial staggered mock community dataset. CPU, central processing unit; GB, gigabyte; RAM, random access memory; Mbp/m, megabase pairs per minute.

Program	number of CPUs	RAM (GB)	Runtime (hr:min:sec)	Classification Speed (Mbp/m)
**Centrifuge**	28	7	0:02:56	1538
**CLARK**	28	297	0:38:40	103
**KrakenUniq**	28	140	2:57:05	22

#### Identification and relative abundance of pathogens in clinical samples

Human samples representing normal blood, endotracheal aspirate of intubated intensive care patients, infected diabetic foot ulcers, and blood from febrile neutropenia patients were sequenced and analyzed with Centrifuge. Table 3 shows the starting number of raw reads, and the percent of total reads classified, unclassified, classified human, and classified non-human by Centrifuge for the 28 samples sequenced.

**Table 3:** Total reads and percent of reads classified by Centrifuge in the human samples. NBD, normal blood donor; wk, week; VAP, ventilator acquire pneumonia; pt, patient; d, day; DFU, diabetic foot ulcer; t, time point; FN, febrile neutropenia.

	raw read number	Centrifuge			
		Classified (%)			Unclassified (%)
		Total (%)	Human (%)	Other organisms (%)	
**NBD 1 wk1**	9,207,771	58.1	53.7	4.4	41.9
**NBD 1 wk2**	2,914,313	31.7	24.9	6.8	68.3
**NBD 1 wk3**	4,504,277	49.9	43.8	6.2	50.1
**NBD 1 wk4**	5,254,026	57.3	54.0	3.3	42.7
**NBD 1 wk5**	2,168,503	67.1	64.4	2.7	32.9
**NBD 1 wk6**	5,269,404	51.2	47.1	4.1	48.8
**NBD 2 wk1**	7,834,015	63.5	60.3	3.2	36.5
**NBD 2 wk2**	5,490,202	30.9	25.5	5.4	69.1
**NBD 2 wk3**	6,705,429	69.1	67.0	2.1	30.9
**NBD 2 wk4**	4,120,356	40.4	35.9	4.5	59.6
**NBD 2 wk5**	2,328,842	61.9	58.8	3.1	38.1
**NBD 2 wk6**	18,302,321	57.0	54.3	2.7	43.0
**VAP pt 1 d1**	3,500,750	92.9	92.5	0.4	7.1
**VAP pt 1 d3**	6,663,668	92.9	92.3	0.6	7.1
**VAP pt 2 d1**	2,719,033	91.3	58.6	32.7	8.7
**VAP pt 2 d3**	2,345,230	92.6	91.8	0.8	7.4
**DFU pt 1 t1**	1,463,794	84.9	84.9	0.1	15.1
**DFU pt 1 t2**	2,753,318	83.1	83.0	0.1	16.9
**DFU pt 1 t3**	3,724,733	84.3	84.1	0.1	15.7
**DFU pt 2 t1**	3,591,666	83.5	82.5	1.0	16.5
**DFU pt 2 t2**	3,097,927	81.3	62.0	19.2	18.7
**DFU pt 2 t3**	2,314,199	89.1	69.7	19.4	10.9
**DFU pt 2 t4**	4,048,863	83.4	62.5	20.9	16.6
**DFU pt 2 t5**	3,225,850	83.5	80.8	2.7	16.5
**DFU pt 2 t6**	2,905,944	84.5	80.6	3.9	15.5
**FN pt 1**	3,497,123	78.8	52.2	26.6	21.2
**FN pt 2**	13,000,518	52.4	35.6	16.8	47.6
**FN pt 3**	18,839,275	64.6	61.1	3.5	35.4

#### Normal blood negative controls identify background false positives and set detection thresholds for clinical samples

**Fig 3:**
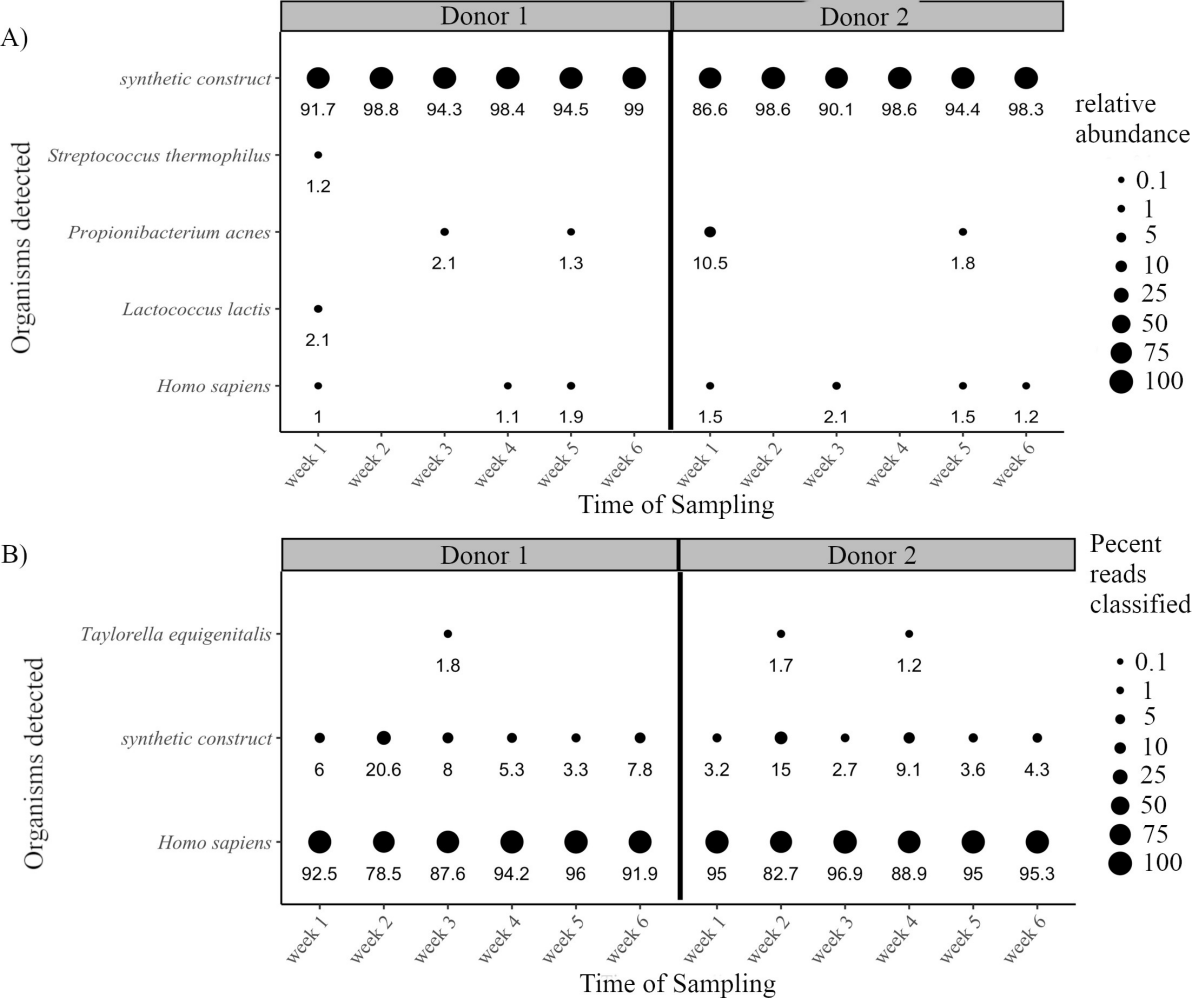
Analysis of background false positives in WGS sequencing from six weekly blood samples of two normal donors. Blood from two normal donors was collected over six weeks, sequenced, and analyzed. A) Results were filtered to include organisms classified at the species or strain-level representing at least 1% relative abundance. Relative abundance of organisms is represented by circle size with actual values displayed below. B) Results were filtered to include organisms with at least 1% of total reads classified. Percent of classified reads is represented by circle size with actual values displayed below.

To provide a negative control for metagenomic sequencing of clinical samples, whole blood was obtained from two healthy donors once a week for six weeks. When the samples were sequenced and analyzed using Centrifuge with a minimum relative abundance threshold of 1%, three skin commensal bacterium *Streptococcus thermophilus*, *Propionibacterium acnes*, and *Lactococcus lactis* were detected along with a synthetic construct (Fig 3A). Due to the extreme difference in genome size between *H*. *sapiens* and synthetic construct, the synthetic construct had high relative abundance despite a low number of reads classified. Thus, while *H*. *sapiens* accounted for 79–97% of the reads classified in the normal blood datasets (Fig 3B), it was calculated to be less than 2% relative abundance by Centrifuge (Fig 3A). Conversely, synthetic construct never accounted for more than 20% of the reads classified (Fig 3B) but had relative abundances of 87–99% after normalization by genome size in the relative abundance calculation (Fig 3A). An additional organism, *Taylorella equigenitalis*, was detected when organisms representing 1% of reads classified were examined. *T*. *equigenitalis* was considered a false positive due to either misclassification or laboratory/reagent contamination due to its appearance in both donors and no prior report of infection in humans. Synthetic construct has been seen in previous metagenomic analyses [38], and the other three bacterium are known to arise in whole blood cultures due to contamination with skin commensals during sample collection. Thus, when the four bacteria above along with the synthetic construct were identified (in all cases as <1% of reads classified) in any subsequent clinical samples, they were excluded. In addition, based on these results from the normal blood samples, subsequent clinical sample results were filtered at 1% relative abundance and 0.01% of total reads classified.

#### Identification of pathogens in ventilator acquired pneumonia

**Fig 4:**
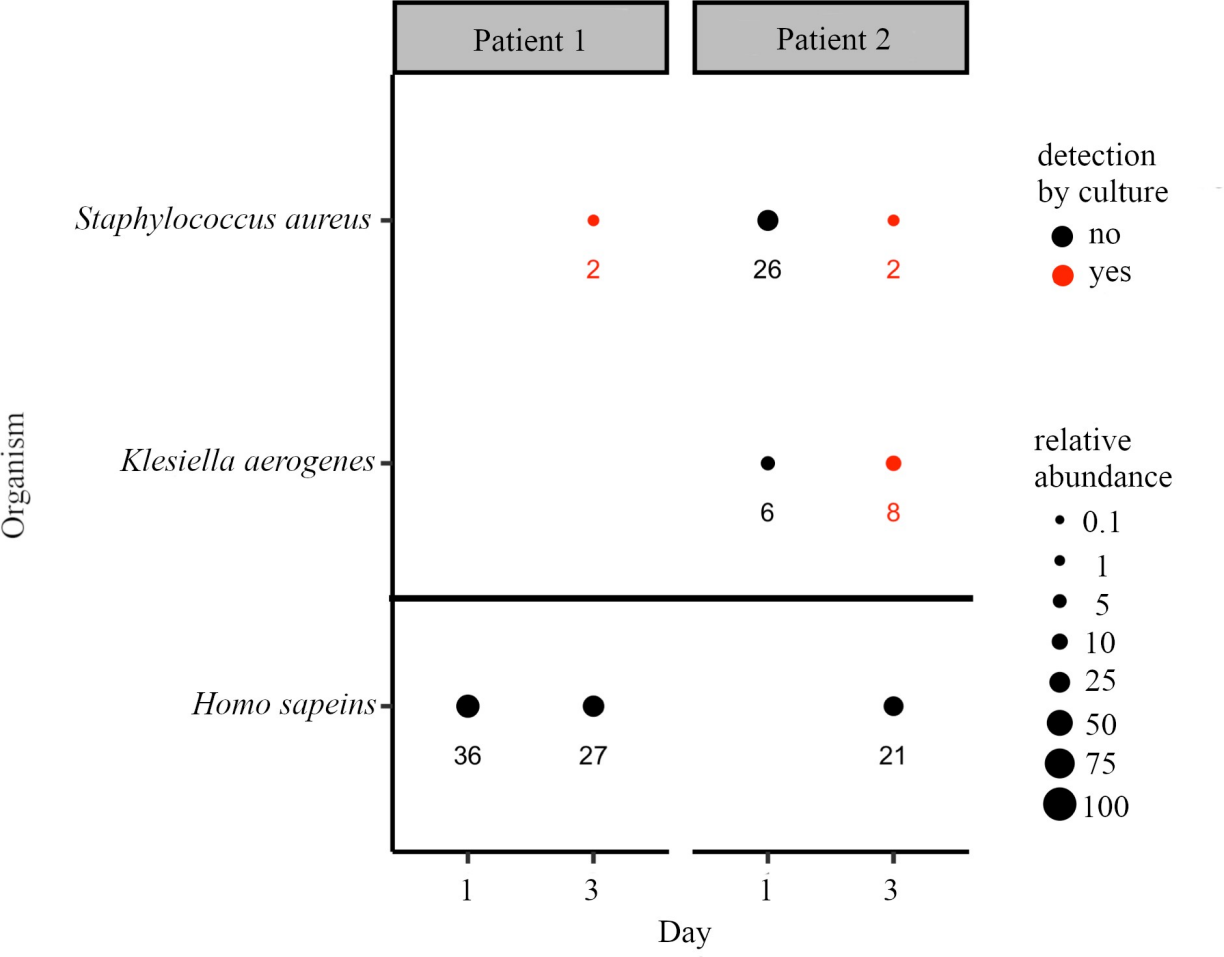
WGS identification and relative abundance of pathogens in patients that developed VAP with comparison to culture. Circle size indicates the relative abundance of the respective organism, with the actual values shown below. Organisms also detected by culture from a BAL on the same day are shown in red and graphed above the horizontal black bar.

Intubated patients were followed longitudinally from the first day of intubation, with samples collected every two days. In contrast to the invasive bronchoalveolar lavage (BAL) used to collect culture samples, samples for WGS were collected from an endotracheal exudate trap attached to the ventilator. Two patients that developed pneumonia were selected for WGS of their samples, and results were compared to culture (Fig 4). On day one of intubation, Centrifuge identified only human reads in the sample from Patient 1. On the third day of intubation, Patient 1 developed pneumonia and culture results from a BAL sample reported heavy growth of methicillin resistant *Staphylococcus aureus* (MRSA). Similarly, *S*. *aureus* was detected in the WGS sample by Centrifuge.

In contrast to Patient 1, Patient 2 was found to have both *S*. *aureus* and *Klebsiella aerogenes* from day one of intubation by WGS (Fig 4 Patient 2). On day three of intubation, the patient was diagnosed with pneumonia, and culture of a BAL sample was strongly positive for MRSA and *K*. *aerogenes*. Both organisms were also detected by WGS in the day 3 endotracheal aspirate sample.

Both VAP patients’ culture results identified not just *S*. *aureus*, but MRSA, raising the question of whether WGS could identify the presence of the *mecA* gene responsible for methicillin resistance in *S*. *aureus*. The presence of *mecA* was detected using the Comprehensive Antibiotic Resistance Database resistance gene identifier tool in the three WGS datasets in which *S*. *aureus* was identified by Centrifuge. The *mecA* gene was detected in only one sample (Patient 2 day 1) which contained 109 reads (of 619,389 reads classified as *S*. *aureus* from 2,719,033 total) that aligned to *mecA*. The 109 reads provided complete coverage of *mecA* at a depth of 2-17x (S1 Fig). The other two samples had too few reads classified as *S*. *aureus* to provide sufficient coverage to detect *mecA*: Patient 1 day 3 had 962 reads classified as *S*. *aureus* with none matching *mecA*, while Patient 2 day 3 had 472 S. aureus reads with none matching *mecA*.

### Identification of pathogens in longitudinal samples of infected diabetic foot ulcers

Patients seen for treatment of diabetic foot ulcers were followed longitudinally. Two patients with infected ulcers that progressed (despite standard of care) to lower limb amputation were selected for WGS analysis and results were compared to culture. DFU samples consisted of curettage samples or necrotic tissue obtained during wound debridement. In three of the four VAP samples analyzed above, human reads constituted >90% of the classified reads (Table 3). In an attempt to reduce human reads in subsequent analyses, a simple sample processing protocol was employed: pathogens were enriched by low speed centrifugations followed by filtering (described in methods). Despite pathogen enrichment steps, the first three DFU samples yielded the lowest number of non-human organisms classified by Centrifuge (Table 3), possibly because the human DNA contamination was not from intact human cells, but instead arose from cell-free DNA released into the wound from necrotic tissue.

**Fig 5:**
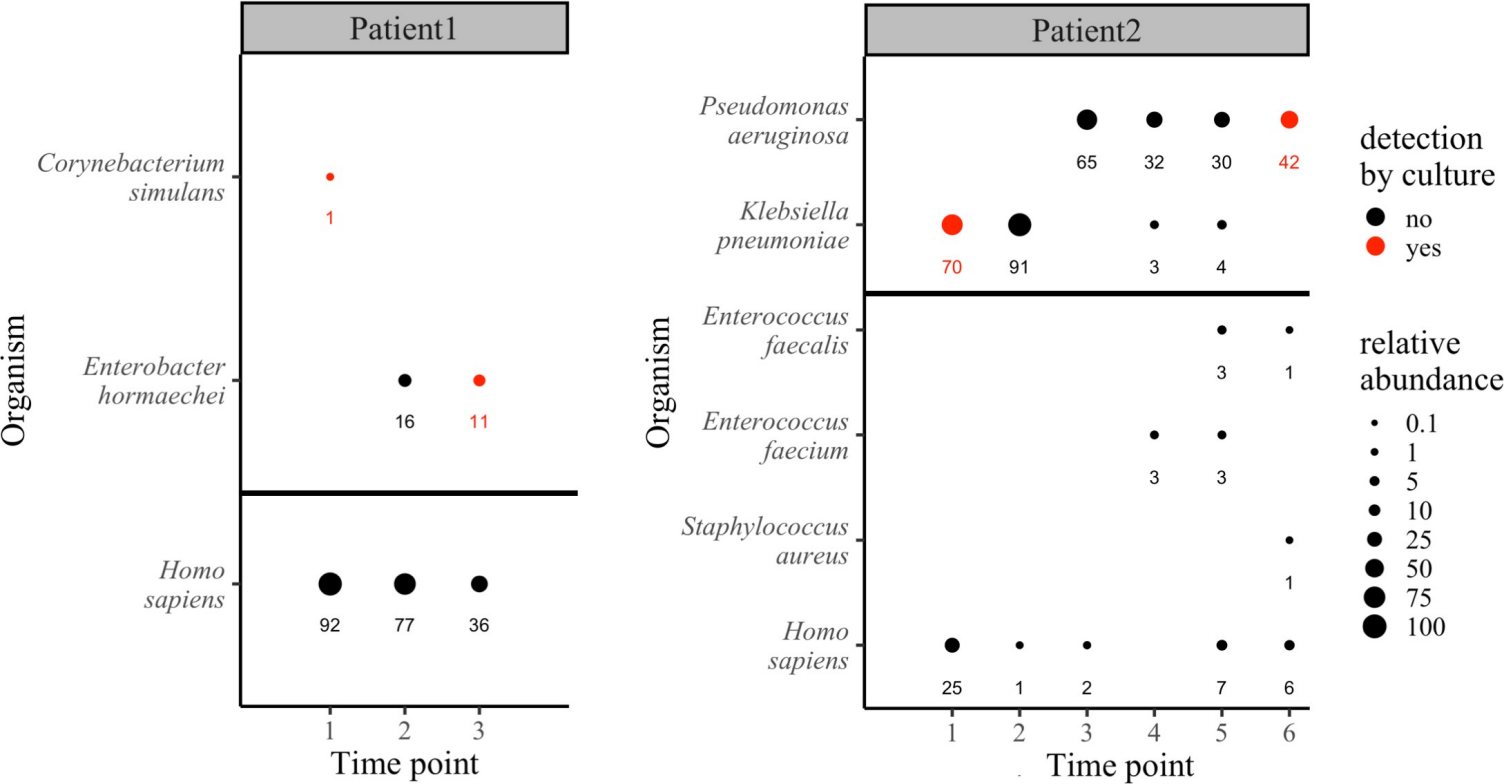
WGS identification and relative abundance of pathogens in longitudinal samples of infected diabetic foot ulcers with comparison to culture. Circle size indicates the relative abundance of the respective organism, with the actual values shown below. Organisms also detected by culture on the same day are shown in red and graphed above the horizontal black bar.

In the first time point for Patient 1, WGS detected a low prevalence of *Corynebacterium* (1% relative abundance), while culture detected *Corynebacterium* (3+), *Streptococcus sp*. (1+), *Haemophilus influenzae* (3+), and mixed flora (3+) (Fig 5). Based on the culture results and clinical signs of infection, broad-spectrum antibiotic therapy was initiated. The wound continued to worsen despite antibiotic therapy, wound debridement, and proper wound care. After six weeks of antibiotic therapy the wound was not healing, and another culture detected only *Corynebacterium* (2+) and *Staphylococcus simulans* (1+). In contrast, WGS revealed a complete conversion of the wound microbiome to 100% *Enterobacter hormaechei*, a member of the *E*. *cloacae* complex [40] (Fig 5). Four weeks later the wound continued to worsen, and the patient developed a fever, precipitating admission to hospital for impending sepsis and re-initiation of antibiotic therapy. A third culture sample was taken upon admission to hospital, this time detecting 3+ *E*. *cloacae* four weeks after WGS had detected it at time point 2. The third WGS analysis at this time again identified *E*. *hormaechei* as the sole organism in the wound (Fig 5). Despite antibiotic therapy, the wound continued to worsen resulting in an amputation to save the patient.

Patient 2 was followed over six time points and presented at the first time point in septic shock. A culture sample identified *Enterococcus faecium* (3+) and *K*. *pneumoniae* (1+), while WGS identified only *K*. *pneumoniae* (Fig 5). Based on culture results, broad spectrum antibiotic therapy was initiated targeting *E*. *faecium*. Three weeks later, the wound had not healed so antibiotic therapy was switched to target *K*. *pneumoniae* (no new culture was taken). The WGS analysis of time point 2 again identified the wound as dominated by *K*. *pneumoniae*. After ten days of the second antibiotic therapy, the wound continued to worsen and a second culture identified only *Corynebacterium* sp. (3+) and *Staphylococcus simulans* (1+), while WGS of this fourth time point indicated the wound microbiome had become completely dominated by *Pseudomonas aeruginosa* (Fig 5). In addition to *P*. *aeruginosa*, two *P*. *aeruginosa*-specific phages were detected by Centrifuge (Pseudomonas phage YMC11-02-R656 and Pseudomonas phage vB_PaeP_Tr60_Ab31, data not shown). Over the next three weeks, cultures at time point 4 and 5 continued to miss *P*. *aeruginosa*, reporting only mixed flora and *Corynebacterium*, while WGS continued to indicate dominance by *P*. *Aeruginosa* and detected re-emergence of *K*. *pneumoniae* (Fig 5). The wound continued to worsen despite a third round of broad-spectrum antibiotics. Culture finally identified *P*. *aeruginosa* at the sixth time point, six weeks after detection by WGS, by which point the patient was again septic and required an amputation.

### Identification of pathogens in whole blood from febrile neutropenia patients

**Fig 6:**
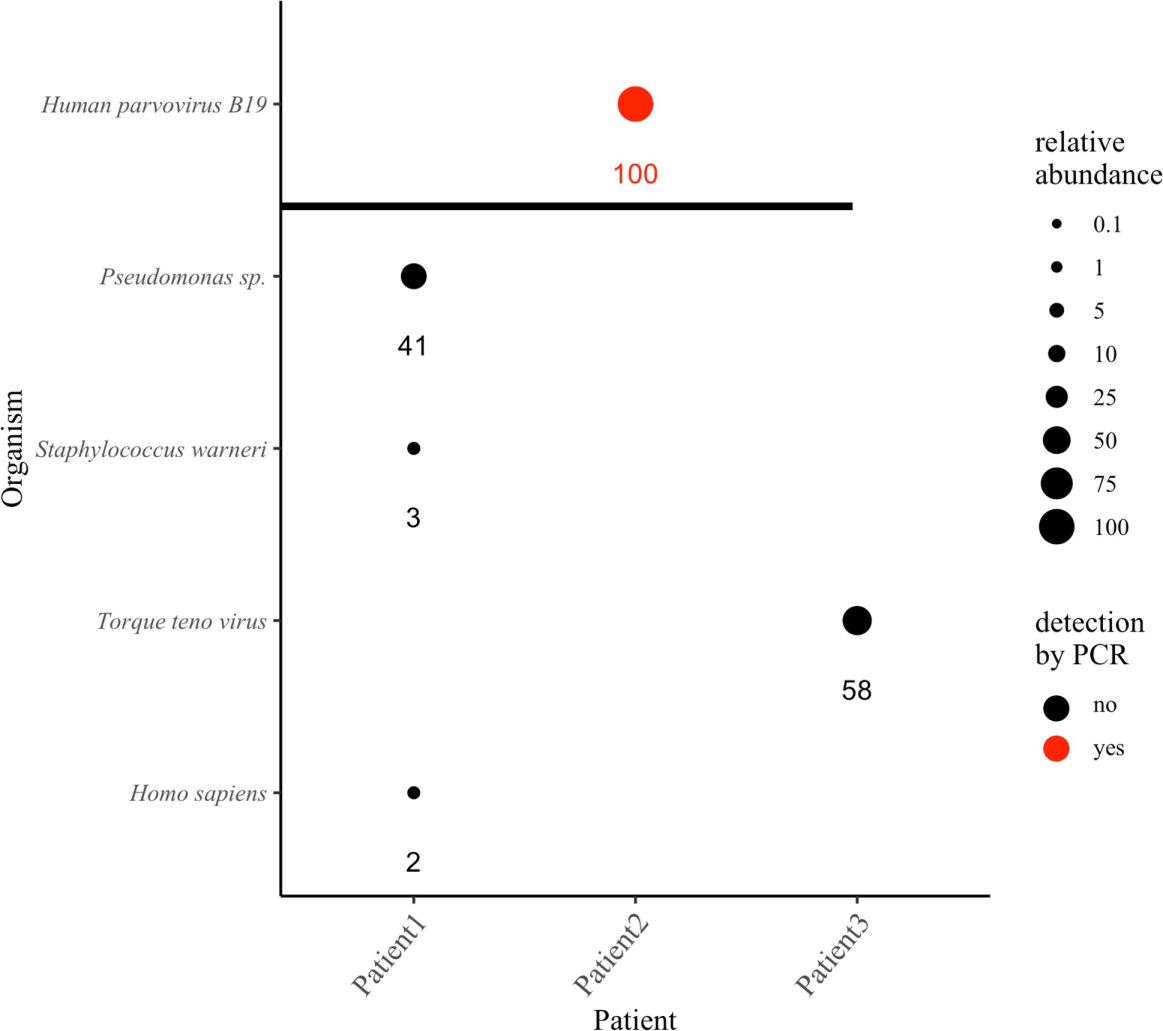
WGS identification and relative abundance of pathogens in febrile neutropenia samples. Circle size indicates the relative abundance of the respective organism, with the actual values shown below. Human parvovirus detected by a clinical PCR assay are shown in red and graphed above the horizontal black bar. The relative abundances of the multiple strains of Torque teno virus and species of *Pseudomonas* classified by Centrifuge were summed and reported as Torque teno virus and *Pseudomonas sp*., respectively.

Three neutropenic leukemia/lymphoma patients self-reported to the University of Arizona Cancer Center clinic with fever and were diagnosed with FN. Blood cultures were ordered, at which time a whole blood sample was taken for WGS analysis. Three likely pathogens were identified by Centrifuge: *Pseudomonas sp*. with a relative abundance of 41% in Patient 1, human parvovirus B19 with a relative abundance of >99% in Patient 2, and Torque teno virus with a relative abundance of 58% in Patient 3 (Fig 6). For the two viruses detected, the number of reads classified to each was small relative to the total, however, as with the synthetic construct in the normal blood controls, Centrifuge compensated for their small genome sizes in the relative abundance estimates. Torque teno virus’s relative abundance was 51%, while representing only 1.6% of the reads classified, while human parvovirus had a relative abundance estimate of >99%, while representing only 13.9% of the reads classified.

Blood culture results for all three patients were negative, both at the time of WGS sample collection and in two subsequent blood cultures of each patient. Thus, the sequencing results could not be corroborated by culture. However, patient two was positive for human parvovirus in a clinical PCR test in the month before and after the WGS sample was obtained, supporting the WGS results. In FN Patient 1, the predominant *Pseudomonas* species identified was *P*. *fluorescens*. *Pseudomonas* has been reported as a false positive in a prior study of laboratory and reagent contaminants, and *P*. *fluorescens* is not generally considered a human pathogen. However, the fact that *P*. *fluorescens* did not appear in the normal control blood samples that used the same laboratory and reagents, and is known to infect immunocompromised individuals [41], suggests the finding in FN Patient 1 is not artifact.

### Genome coverage of suspected pathogens in febrile neutropenic patients

**Fig 7:**
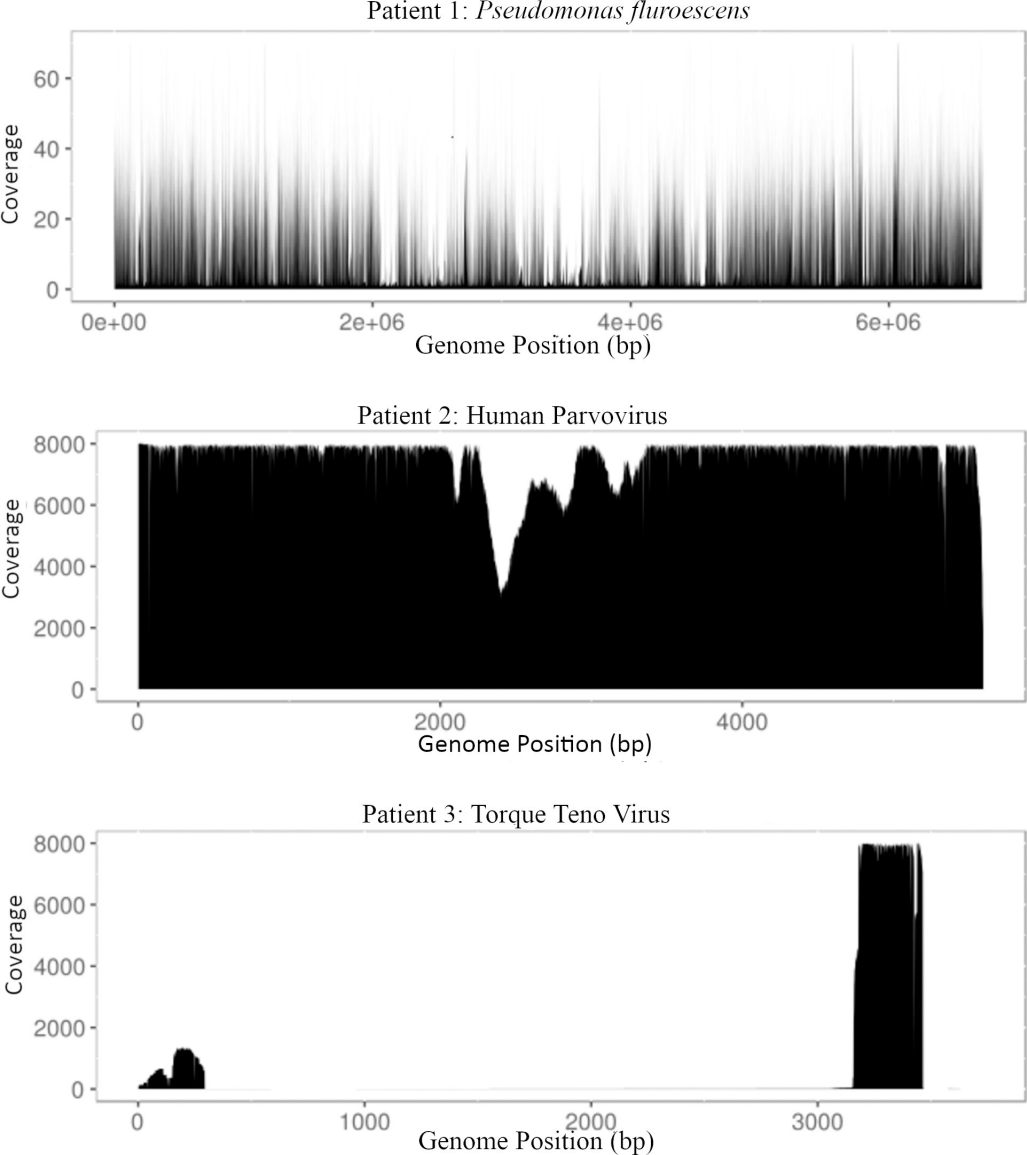
Genome coverage of suspected pathogens identified in febrile neutropenia patients. For each patient, reads were aligned to the reference genomes of the most likely pathogen identified by Centrifuge and read coverage at each base graphed relative to the position in the respective genomes.

Reads from the three FN samples were aligned to the respective reference genomes of the suspected pathogens to determine average depth of coverage (Fig 7). When Patient 1 reads were aligned to the *Pseudomonas fluorescens* genome, the average coverage was 7.0. Patient 2 reads aligned to the human parvovirus B19 genome showed average coverage of 5,180. Finally, Patient 3 reads aligned to the Torque teno virus (TTV) genome showed high coverage (~8,000) for a ~500 base pair region of the genome.

### Effect of quality control and to host read removal on taxonomic assignment and relative abundance estimates

Quality control of sequence reads and removal of reads that align to the host genome are commonly done before taxonomic classification, adding to analysis time. We tested the effect of not performing quality control of sequence reads before Centrifuge analysis on the staggered mock bacterial community and the FN patient datasets. Eliminating quality control caused a single, low abundance false positive classification in the mock community, while causing viral reads to be preferentially removed (S1 File). Consequently, quality control was not performed for clinical sample analyses.

We next compared three methods of removing host (human) reads on the taxonomic classification and relative abundance estimates of the FN samples (S2 File). All three resulted in additional low abundance organisms being identified by Centrifuge. These organisms are likely spurious due the fact that removal of human reads greatly affected the percent of total reads that were classified to these organisms, and thus caused them to pass the filters set by the normal blood negative controls. Consequently, host read removal was not performed for any of the analyses reported.

## Discussion

The first dataset used to assess the three classifiers was a series of binary bacterial mixtures chosen for their phylogenetic distance, and mixed so that each pair was combined across three logs of relative abundance. Centrifuge was able to discriminate the most closely related pair of bacteria, *E*. *coli* and *S*. *flexneri*, even when one of the organisms was present as 0.1% of the mixture while KrakenUniq and CLARK failed to detect the lowest relative abundance of *S*. *flexneri*. As the proportion of *E*. *coli* decreased, the relative abundance estimates for all three classifiers diverged from expected, so that the *E*. *coli* estimate was >1% when *E*. *coli* was only 0.1% of the mixture. The same inaccuracy did not occur as the *S*. *flexneri* relative abundance decreased to 0.1%, suggesting Centrifuge misidentified a portion of the *S*. *flexneri* genome as *E*. *coli* but not the other way around, while KrakenUniq and CLARK failed altogether. The difficulty classifying *S*. *flexneri* was further suggested by the fact that the false positive rate increased as *S*. *flexneri* relative abundance increased. One possible cause for more relative matches to *E*. *coli* than *S*. *flexneri* is that *E*. *coli* strains and isolates represent the most substantial fraction of the reference database. Another possibility is false positive identification of reads as *E*. *coli*, for example, McIntyre et al. [31] saw similar false positive identification of *E*. *coli* when using metagenomic classifiers on negative control sequences not belonging to any known organism. Although Centrifuge uses a modified FM-index to condense closely related genomes, the total file size of base pairs maintained (unique + shared based on ≥ 99% identity) exceeds the relative file size of all other species [34] giving it a higher probability for matches. Centrifuge’s better performance with the *E*. *coli*/*S*. *flexneri* mixture suggests that Centrifuge’s use of a modified FM-index dampens the effect of multiple strains and isolate genomes, but the effect is still present for highly abundant organisms such as *E*. *coli*. In contrast to Centrifuge and CLARK’s abundance estimates, KrakenUniq classified the majority of *S*. *flexneri* and *E*. *coli* reads to the family (*Enterbacteriacae*) level, only estimating 11.6% relative abundance of S. flexneri when it was in fact 99.9% of the sample. KrakenUniq’s assignment of the majority of the *S*. *flexneri*/*E*. *coli* reads to a higher taxonomic level results from its strategy for taxonomic assignment of reads. Specifically, reads from closely related organisms in which a read that could be assigned to multiple species are instead assigned to the nearest common taxonomic level. Therefore, the KrakenUniq abundance estimates are not strictly comparable to CLARK and Centrifuge without further analysis and re-calibration.

Interestingly, phylogenetic distance did not predict the accuracy of Centrifuge and CLARK’s relative abundance estimates. Both were capable of reliably detecting organisms down to 1% abundance, regardless of phylogenetic distance. A reasonable assumption would be that as phylogenetic distance increases, the number of discriminatory k-mers would also increase and allow better classification of reads. Instead, both Centrifuge and CLARK had better sensitivity to the lowest relative abundance in the most closely related pair (*E*. *coli*/*S*. *flexneri*) than the intermediate pair (*S*. *pyogenes*/*S*. *saprophyticus*) where both organisms only shared the same phylogenetic class (*Bacilli*). Overall, while Centrifuge and CLARK out-performed KrakenUniq in terms of relative abundance accuracy, CLARK proved the most accurate due to Centrifuge’s difficulty with correctly estimating *S*. *pyogenes* in the most distant phylogenetic pairing.

One drawback of using Centrifuge for clinical pathogen identification is that Centrifuge separates strain-level counts, splitting reads among species strains, which required manually summing strain level abundances for reporting. Future iterations of Centrifuge could address this issue by re-analyzing the data with a reduced reference set of genomes based on the first round of analysis or using a reduced reference database. Lastly, current reference databases do not account for all of the extant microbial/viral diversity that may be present in patients as evidenced by *A*. *odontolyticus’* absence from the default Centrifuge and KrakenUniq databases. However, this issue can be addressed by database curation and the exponential growth in the number of microbial draft genomes available [42].

In addition to KrakenUniq’s trouble separating clinically important bacteria, and KrakenUniq’s and CLARK’s reporting of false positives in the mock community, the processing time and computational resources required by KrakenUniq and CLARK were far greater than Centrifuge (Table 2). Therefore, we focused on Centrifuge in the subsequent clinical analyses. The much larger RAM requirements for KrakenUniq and CLARK relative to Centrifuge (Table 2) are consistent with a previous comparison of Kraken and Centrifuge (Kim et al. 2016), and the study in which McIntyre *et al*. [31] reported CLARK’s RAM requirements were similar to Kraken’s. Given the greatly reduced computer system requirements for Centrifuge, analyses could run on a well-equipped personal computer (e.g. 18-core processors and 8GB RAM) compared to CLARK and KrakenUniq that require a server (e.g. 28-core processors and > 256GB RAM) to achieve comparable speeds to those reported in Table 2. Future adoption of WGS approaches will be constrained by the total time from sample collection to delivery of results. This time could be reduced to 6–12 hours with current technology, thus putting a premium on the data analysis steps being as rapid as possible, preferably requiring only minutes to perform. The reduction in time and resources achieved by Centrifuge relative to the other taxonomic classifiers likely owes to its compression of the reference database [34]. Thus, despite Centrifuge’s drawbacks such as separation of results to strain level and reporting phages separately from their hosts, Centrifuge's lower false positives, greater speed, and lower memory requirements suggests it may be a good starting point for clinical applications.

### Normal whole blood for false positive detection and background thresholds

Six longitudinal whole blood samples from two donors were processed to deplete human cells (and thus human sequence reads in the datasets) by centrifugation and filtration similar to an approach that has been recently published [43]. Despite the efforts to reduce human reads, the majority of classified reads were still human (normal blood donors, Table 3), presumably because there was low biomass from other organisms in the normal blood as would be expected from such a sterile site. Despite well-documented issues with spurious results arising from reagent and laboratory contamination [27], when organisms with relative prevalence below 1% were ignored, no credible organisms other than *Homo sapiens* were detected in normal blood. In addition, the three organisms that were identified in the normal whole blood (Fig 3A) are all recognized skin commensals that are also detected by culture as a result of sample collection. Despite the possible advantages WGS over culture, skin contaminants will continue to present themselves since the whole blood sample collection method is the same. The approach of ignoring low prevalence organisms is a simple means of reducing spurious results, while other physical and statistical approaches have been developed [44,45] and will be of importance to developing reliable clinical tests in the future. Our results with normal blood stand in contrast to a much larger study [46] in which normal blood was consistently found to contain predominantly *Sphingomonas* bacterial DNA. We note, however, that S*phingomonas* was found to be a consistent and significant contaminant of PCR master mix [44], possibly explaining the detection of *Sphingomonas* in so many normal blood samples. Suppression and identification of spurious results, which appear to be much more likely when actual sample pathogen biomass is low or negative, (e.g., a patient with a fever is suspected of bloodstream infection but is actually febrile due to chemotherapy), will be a critical component of clinical applications of metagenomic sequencing.

### Pathogen detection in clinical samples

Sixteen samples from seven patients were sequenced by WGS and analyzed with Centrifuge. In both VAP patients, culture samples were taken when pneumonia was diagnosed on day three, and all three organisms identified by culture were identified by WGS, despite the sample types being different (BAL versus endotracheal aspirate). The ability of WGS to detect the culture-identified pathogens from a non-invasive sample suggests the possibility of WGS-based surveillance of intubated patients to detect emergence of infection before pneumonia symptoms arise.

In the first DFU patient, culture initially detected a number of specific organisms (*Streptococcus sp*., *H*. *influenzae*, *Corynebacterium*) and a vague number of others (“mixed flora”) while WGS only detected *Corynebacterium*. Following a course of broad-spectrum antibiotics, culture failed to identify the complete conversion of the wound to a single organism (*E*. *Hormaechei*, a member of the *E*. *cloacae* complex) that was detected by WGS. It was not until the third time point that culture identified *E*. *cloacae*, by which time the wound had progressed to the point that amputation was required. There was a similar conversion of the wound microbiome in Patient 2, in whom culture initially identified *E*. *faecium* and *K*. *pneumoniae*, while WGS only identified *K*. *pneumoniae*. Following a course of antibiotics targeting *E*. *Faecium*, the wound underwent a complete conversion to *P*. *aeruginosa* that was missed by culture until six weeks later, by which point the patient required an amputation. While both wounds were clinically infected from the start of observation, both underwent a profound change in their microbiomes that was detected by WGS weeks before culture. The drastic changes in the wound microbiomes were presumably a result of the antibiotic therapy initiated after the first culture results, with the wounds in both cases becoming dominated by organisms that were not susceptible to the antibiotics used. The results from the two DFU patients point to the possibility of improved wound surveillance by WGS.

Lastly, Torque teno virus was identified in a cancer patient undergoing bone marrow ablation in preparation for a hematopoietic stem cell transplant. This finding highlights the possible value of the metagenomic sequencing approach as Torque teno virus has been investigated as a predictive marker for post-transplant complications [47]. In addition, Torque teno virus was also reported recently in a prospective study of the utility of WGS analysis in cerebrospinal fluid samples. The identification of Torque teno virus and human parvovirus (which was corroborated by a clinical PCR assay) in the FN patients indicates that the sample preparation methods used in this study can isolate and detect viruses.

### Identification of a drug resistance gene in a clinical sample

Bacterial drug resistance is a serious worldwide human health problem [48]; and its detection in clinical samples can be as important as a species-level identification. For example, detection of “methicillin-resistant gram-positive cocci” by culture is more relevant to clinical decision making than a detection of *S*. *aureus* by WGS that misses the presence of *mecA*. To this end, we attempted to identify *mecA* in the three samples that were positive for *S*. *aureus* by WGS from the two VAP patients diagnosed with MRSA. Low coverage in two of the samples prevented detection *mecA*, but the sample from Patient 2 on day 1 of intubation had enough reads that the entirety of the *mecA* was covered. Interestingly, no culture sample was taken on day one, as the patient was not diagnosed with pneumonia until day three. Thus, WGS identification of MRSA on day one, two days before diagnosis of pneumonia, and from a non-invasive sample, supports the potential for WGS surveillance of intubated patients before symptoms of pneumonia arise. Also of note, was the ability of WGS to detect *S*. *aureus* even in the other two samples with low numbers of *S*. *aureus* reads. For example, in the sample from which *mecA* was detected, the *S*. *aureus* relative abundance was 26% calculated from 619,389 reads classified as *S*. *aureus* out of a total of 2,719,033. In contrast, the day three relative abundance (when pneumonia was diagnosed, and MRSA detected by culture) was 10% calculated from just 962 reads classified as *S*. *aureus* out a total of 2,345,230. A recent prospective study using WGS and the same drug resistance identification tool used here was also successful in detecting drug resistance genes in samples of cerebrospinal fluid [24]. Given the critical importance of drug resistance to the success of antibiotic therapy, the interplay between total number of reads obtained, number of reads classified to a pathogen, number of reads classified to the host genome, and the relative genome sizes involved will need to be carefully explored for successful clinical translation of WGS.

### Genome coverage of presumptive pathogens identified in FN patient samples

We examined the genome coverage of the pathogens identified in the FN patients with the assumption that the genomes of the pathogens should be represented by consistent coverage, whereas uneven coverage could indicate insufficient evidence of organism presence. Parize et al. [29] took a similar approach in which even distribution of contigs was used as part of the criteria to decide if a sample was deemed positive [29]. Interestingly, Torque teno virus sequence found in Patient 3 had high coverage of only a ~500 base pair untranslated region of the genome. This highly conserved region has been suggested to be critical for viral replication, and may indicate an early replication event or the presence of sub-viral particles, a characteristic that has previously observed in Torque teno virus [49]. The evidence for sub-viral particles provided by the coverage analysis is the first from an *in vivo* sample.

### Host screening and quality control

Although quality control of raw reads is imperative for variant calling and genome assembly, it takes considerable computing time and resources. In this study, we observed limited benefits of quality control regarding accurately identifying and quantifying the abundance of the bacteria in the staggered mock community (S1 File, Fig A). Although quality control of reads eliminated a single false positive organism estimated at 2.3% relative abundance, quality control of reads from the FN data showed a bias toward removing viral reads (S2 File, Table 1). The limited effect of quality control on Centrifuge’s performance likely stems from the overall high quality of base calls on the Ion Torrent sequencer within the context of k-mer based classifiers. Clinical development of a tool such as Centrifuge will have to weigh the limited benefits of quality controlling data before analysis versus the bias toward the removal of viral reads and the time required to perform quality control.

Despite efforts to enrich microbial/viral DNA by centrifugation and filtering, a large proportion of reads were still classified as human in the clinical samples, especially in the DFU samples where extracellular human DNA would be expected due to necrosis (Table 3). Screening host reads by alignment to the human reference genome appears to be unnecessary and even detrimental as it caused additional likely spurious organisms to pass filtering (S2 File). Given that reference genomes can contain sequences of mixed origin due to horizontal gene transfer, endogenous and integrated microbes/viruses, prophage in bacterial genomes, as well as library preparation and sequencing contamination, classifying reads without host screening appears to be the best compromise between preserving accuracy and the reduced speed of analysis.

### Conclusion

In summary, our analyses suggest that Centrifuge, an open-source software for fast taxonomic classification, provides accurate identification and abundance estimates in clinically relevant metagenomes, while more efficiently using computational resources and time relative to competing tools. Centrifuge's ability to quickly assign taxonomy to reads, accurately represent the abundance of organisms such as viruses, and sidestep read quality control and host-screening make it a good candidate for classifying reads of clinically relevant organisms. Consequently, we have made Centrifuge and the bubble plot software used in the study available as Apps in iMicrobe (http://imicrobe.us) to provide public streamlined access.

## Materials and methods

### Reference datasets for taxonomic classification benchmarking and analysis

#### Binary bacterial mixtures

The binary mixtures were described previously [18]. Briefly, four species of bacteria were used to create three binary mixtures representing: (1) difficult to discriminate species with divergent clinical impact (*Escherichia coli* versus *Shigella flexneri*), (2) Gram-positive species (*Staphylococcus saprophyticus* versus *Streptococcus pyogenes*), and (3) Gram-positive versus Gram-negative species (*E*. *coli* versus *S*. *saprophyticus*). DNA from the bacteria were purchased from the American Type Culture Collection (Manassas, VA, USA) and mixed in pairs so that each species represented 99.9, 99, 90, 50, 10, 1, and 0.1% of the total sample. Samples were sequenced as described below, and the sequence data deposited to the NCBI Sequence Read Archive under accessions: SRX3154186-SRX3154219 in project accession PRJNA401033.

#### Staggered mock bacterial community

The staggered mock bacterial community (BEI Resources, Manassas, VA, USA, Microbial Mock Community B HM-277D) consists of 20 bacterial species mixed to provide specific 16S rRNA gene copy numbers for each species. Using the 16S rRNA gene copy numbers, along with the known 16S rRNA gene copy number in each species’ genome, we calculated the number of genomes present for each species to provide an expected value for comparison to the relative abundance estimates of the classifiers. The mock community was sequenced as described previously [8] and sequence data deposited to the NCBI Sequence Read Archive under accession: SRP115095 in project accession PRJNA397434.

#### Read quality control

To ensure that only high-quality reads were used to compare the three classification tools, all reads from the binary bacterial mixtures and staggered mock community were converted to FASTQ format from raw BAM files with BEDtools’ bamtofastq v2.17.0 [50]. FastX toolkit v.0.0.14 (http://hannonlab.cshl.edu/fastx_toolkit), was used to perform quality control measures on FASTQ data including quality filtering (using parameters -q 17 -p 80), trimming and setting a minimum read length (using parameters -f 10 -l 175), and removal of short reads (using parameter -l 50) and duplicate reads. The scripts to quality control data are available at: https://github.com/hurwitzlab/pathogens_in_clinically_relevant_samples/tree/master/0_pre-processing_scripts

### Benchmarking reference datasets

#### CLARK read classification

CLARK v1.1.3 [32] was used to classify reads to known taxa using the default CLARK database (downloaded on March 3, 2016). Reads were classified using the classify metagenome command, a mode of 1, and k-mer size of 31. Next, the abundance of organisms was estimated using the estimate_abundance command with default settings. Abundance reports were filtered to include only species with a minimum of 0.1% abundance (bacterial mixtures) or 0.01% (staggered mock community) of the classified reads.

#### Centrifuge read classification

Centrifuge v1.0.3-beta [34] was used to classify reads to known taxa. Each of the analyses below were run using default parameters.

Binary bacterial mixtures analysis: Centrifuge was run with the “p_compressed+h+v” database at http://www.ccb.jhu.edu/software/centrifuge/ (last updated 12/06/2016).

Centrifuge abundance report results were filtered to include only organisms at the species or strain-level with a minimum of 0.1% of total reads classified. False positives were calculated by summing the relative abundances of any organism classified by Centrifuge that were not part of the mixture.

Staggered mock bacterial community analysis: Because the staggered mock community included *Actinomyces odontolyticus*, and this organism was not present in the recommended default Centrifuge database (p_compressed+h+v), the mock community was analyzed using a custom database generated from 23,276 complete archaeal, bacterial, and viral genomes. The database was downloaded from Refseq on July 2017 using the centrifuge-download and centrifuge-build scripts respectively. The composition of the custom database is available at https://github.com/hurwitzlab/pathogens_in_clinically_relevant_samples/tree/master/0_custom_centDB. Centrifuge abundance report results were filtered to include only organisms at the species or strain-level with a minimum of at least 0.01% abundance as calculated by Centrifuge with no minimum number of reads. Classifications to strains or subspecies were summed at the species level, while hits to phages were ignored.

#### KrakenUniq read classification

KrakenUniq v0.5.8 was used to build a reference database using the default parameters (script available at https://github.com/hurwitzlab/krakenuniq/blob/master/scripts/build-ocelote.sh). KrakenUniq was then run using default parameters, at the “species level” (https://github.com/hurwitzlab/krakenuniq/blob/master/scripts/run.sh) on the reference datasets. Abundance reports were filtered to include only species with a minimum of 0.1% abundance (bacterial mixtures) or 0.01% (staggered mock community) of the classified reads.

#### Benchmarking

Centrifuge, CLARK and KrakenUniq computational requirements were assessed on the staggered mock community after quality control (size of the file: 4 gigabytes). CLARK and KrakenUniq were run on a high-performance computer cluster with exclusive access to a high-memory node (total size 28 cores (Xeon Broadwell E5-2695 Dual 14-core processors) and 2TB total memory); whereas Centrifuge was run on a standard node because of the lesser memory requirements (total size 28 cores (Xeon Broadwell E5-2695 Dual 14-core processors) and 192 GB total memory). Wall clock time was computed using the GNU `time`command, memory consumed was reported by the distributed computing software PBS Pro `tracejob`command.

#### Centrifuge and KrakenUniq benchmarking with 31 datasets

To place Centrifuge within the context of the McIntyre and Breitwieser studies [31,38], Centrifuge and KrakenUniq were compared using 10 biological and 21 simulated datasets from Breitwieser et al. [38]. The performance of each classifier was measured by calculating the recall (true positives / (true positives + false negatives)) and F1 score (2*(precision*recall)/(precision+recall)) for the detection of organisms at the species level. Results were filtered using the relative abundance threshold reported as “minimum abundance threshold” in S1 Table.

### Clinical datasets

#### Ethics statement

The Institutional Review Board at the University of Arizona approved the human subjects research for the FN and DFU samples, and HonorHealth Scottsdale Institutional Review Board approved the human subjects research for the VAP samples. Informed consent was obtained from FN and DFU patients, while the passive nature of the VAP sample collection did not require informed consent.

#### Patient sample preparation and processing

Normal blood: whole blood was collected and subjected to pathogen isolation and DNA isolation in the same manner as the FN samples (see below), and the resulting DNA sequenced. Blood was obtained from two healthy donors once a week for six weeks and processed immediately.

VAP: Endotracheal aspirate (~1–5 ml) was longitudinally collected every 48h starting from time of intubation. Samples were placed in sterile 15 ml tubes and immediately frozen at –80°C until DNA isolation. DNA was isolated from the endotracheal aspirate obtained on days one and three (when both patients were diagnosed with pneumonia and BAL samples were taken for culture) of intubation with the QIAamp BiOstic Bacteremia DNA kit (Qiagen Inc., Germantown, MD, USA) and quantitated on a NanoDrop ND-1000 spectrophotometer (Thermo Fisher Technologies Inc., Santa Clara, CA, USA). DNA was diluted to one ng/ml, and 10 ng used to prepare sequencing libraries.

DFU: Curettage or necrotic tissue samples were collected during wound debridement for DFU patients during standard of care and placed in sterile sample collection containers. Samples were transferred to sterile 15 ml conical tubes (Fisher Scientific, Hampton, NH, USA), and 1 ml of sterile PBS was added followed by vortexing for 30 s. The liquid portion of the sample was drawn off followed by a human cell depletion protocol including three low speed centrifugations 50, 100, and 150 x g for 5 m, 5μm filtering, and a final 4000 x g centrifugation. DNA was isolated from any material sedimented during the final 4000 x g centrifugation with a UCP Pure Pathogen kit (Qiagen Inc., Germantown, MD, USA). Isolated DNA was quantitated on a NanoDrop ND-1000 spectrophotometer (Thermo Fisher Technologies Inc., Santa Clara, CA, USA), diluted to one ng/μl, and 10 ng used to prepare sequencing libraries.

FN: Approximately 5 mL of whole blood were collected (K_2_EDTA BD Vacutainer tubes, catalog #367863 BD Biosciences, San Jose, CA, USA) when blood cultures were ordered for each FN patient and transferred for processing within 2h of collection. Blood samples were diluted with an equal volume of sterile phosphate buffered saline, layered on Ficoll-Paque (GE HealthCare Life Sciences, Pittsburgh, PA, USA) and centrifuged for 20 minutes at 400 x g. Plasma was carefully drawn off, sacrificing some yield to prevent drawing up monocytes, and centrifuged three more times at 50, 100, and 150 x g for 5 minutes to further remove human cells. The plasma was passed through a five-micron filter, and finally centrifuged at 4000 x g to collect microbes. DNA was isolated from any material sedimented during the final 4000 x g centrifugation with a UCP Pure Pathogen kit (Qiagen Inc., Germantown, MD, USA). Isolated DNA was quantitated on a NanoDrop ND-1000 spectrophotometer, diluted to one ng/μl, and 10 ng used to prepare sequencing libraries.

#### DNA library preparation and sequencing

DNA libraries were prepared and sequenced for all samples using Ion Torrent reagents and the Ion Torrent Proton sequencer (Thermo Fisher Technologies Inc., Santa Clara, CA, USA). 10 ng of DNA was used as input to the Ion Xpress Plus Fragment Library Kit (manual #MAN0009847, revC). DNA was sheared using the Ion Shear enzymatic reaction for 12 min, and Ion Xpress barcode adapters were ligated following end repair. Resulting libraries were amplified using the manufacturer supplied library amplification primers and recommended conditions. Amplified libraries were size selected to approximately 200 base pairs using E-gel SizeSelect Agarose cassettes (Invitrogen, Carlsbad, CA, USA) as outlined in the Ion Xpress manual and quantitated with the Ion Universal Library quantitation kit. Equimolar amounts of the library were templated with an Ion PI Template OT2 200 kit V3. The resulting templated beads were enriched with the Ion OneTouch ES system and quantitated with the Qubit Ion Sphere Quality Control kit on a Qubit 3.0 fluorimeter (Qubit, NY, NY, USA). Enriched, templated beads were loaded onto an Ion PI V2 chip and sequenced according to the manufacturer's protocol using the Ion PI Sequencing 200 kit V3. Data were processed with Ion Torrent Server software v4.4.3 to produce data files in BAM format.

#### Clinical dataset availability

Sequence data for VAP, DFU, and FN patient samples were deposited to the NCBI Sequence Read Archive in project accession numbers PRJNA555076, PRJNA554856, PRJNA521396.

### Analysis of clinical datasets using Centrifuge

The datasets were analyzed without quality control or human read removal using the Centrifuge “p_compressed+h+v” database provided at: http://www.ccb.jhu.edu/software/centrifuge/ (last updated 12/06/2016).

Centrifuge abundance report results were filtered to include only organisms at the species or strain-level with a minimum of 0.01% of total reads classified and at least 1% relative abundance. Classifications of synthetic constructs, phages, *Streptococcus thermophilus*, *Lactococcus lactis*, *Propionibacterium acnes* and *Tayorella equigenitallis* identified in normal blood controls were not included in results.

### Effect of quality control and host read host sequence removal on taxonomic assignment

The staggered mock community and FN samples were analyzed before or after quality control. Centrifuge abundance report results were filtered to only include organisms with a minimum of at least 0.01% relative abundance with no minimum percent of reads classified (Staggered mock community) or 1% relative and 0.01% of classified reads (FN samples). Classifications to strains or sub-species were summed at the species level and phages were ignored. Scripts are available at https://github.com/hurwitzlab/pathogens_in_clinically_relevant_samples/tree/master/0_pre-processing_scripts.

The effect of host sequence removal was investigated using the FN samples. Three conditions were tested: 1) alignment to the human genome and removal of aligned reads from the dataset. To remove host (human) reads, FASTQ read files were mapped to HG38 (Genome reference consortium human genome build38) using Bowtie2 [51] and the—very-sensitive option (the script is available at https://github.com/hurwitzlab/pathogens_in_clinically_relevant_samples/tree/master/0_pre-processing_scripts), (2) removing the human sequence from the reference database, and (3) using the "exclude TaxID" function in Centrifuge to exclude reads from classification whose best match was to the human genome. Centrifuge results were filtered to include only organisms with at least 1% abundance and at least 0.01% of classified read. Classifications to strains or sub-species were summed at the species level and phages were ignored.

### Detection and analysis of m*ecA* gene in VAP patient samples

Reads were aligned to the human genome (GRCh37 with contigs) by the Ion Torrent Software Suite (v5.4). Reads that did not align to the human genome in the three VAP BAM files were extracted with samtools (v1.2) and FASTA sequences were analyzed using the Resistance Gene Identifier (RGI) tool available online from the Comprehensive Antibiotic Resistance Database (http://github.com/arpcard/rgi) using default parameters. When *mecA* was indicated by the RGI tool in the Patient 2, day 1 sample, 109 reads matching the *mecA* gene (NG_047936.1) were identified with blastn (v2.7.1) with default parameters. The 109 reads were subsequently aligned to the *mecA* gene with BWA 0.7.17 and coverage data generated with the samtools (v1.2) depth tool to generate coverage values that were graphed in R v3.1.1 (R scripts: https://github.com/hurwitzlab/pathogens_in_clinically_relevant_samples/tree/master/S1_Fig).

### Genome coverage of suspected pathogens from febrile neutropenia patient samples

Reads from FN samples were quality controlled as described above for the bacterial mixtures and staggered mock bacterial community. Following quality control, Bowtie2 [51] was used to map FASTQ reads (with option—very-sensitive) to reference genomes for the organisms identified by Centrifuge (*Pseudomonas fluorescens* accession: NC_012660.1, human parvovirus B19 accession: NC_000883.2, Torque teno virus accession: NC_015783.1). Coverage data was generated with samtools (v1.3.1, [52]) depth tool from the resulting BAM files and visualized in R v3.1.1 (R scripts are available at: https://github.com/hurwitzlab/pathogens_in_clinically_relevant_samples/tree/master/7_depth_coverage).

### Software availability

To improve access to Centrifuge and the bubble chart visualizations used in this manuscript, both have been made available on iMicrobe (https://www.imicrobe.us). As a starting point, researchers may run centrifuge-1.0.4u2 (https://www.imicrobe.us/#/apps/centrifuge) followed by centrifuge-bubble-0.0.5u1 (https://www.imicrobe.us/#/apps/centrifuge-bubble). Source code for running Centrifuge on a high-performance compute cluster, using a singularity image and scripts for formatting the results are available at: https://github.com/hurwitzlab/centrifuge.

Source code for running KrakenUniq on a high-performance compute cluster, using a singularity image (KrakenUniq v0.5.8) and scripts for formatting the results are available at: https://github.com/hurwitzlab/krakenuniq.

Analyses, scripts and visualizations shown in the manuscript are archived at: https://github.com/hurwitzlab/pathogens_in_clinically_relevant_samples.

## Supporting information

S1 Table Centrifuge and KrakenUniq F1 and recall scores for 31 benchmarking datasets. 31 “truth sets” previously analyzed previously by McIntyre et al. [31] and Breitwieser et al. [38] for evaluation of metagenomic classifiers were analyzed with KrakenUniq and Centrifuge to place Centrifuge within the context of the prior studies. F1 and recall for the biological and *in silico* datasets are shown at the species level.
(XLSX)

S1 Fig The *mecA* gene coverage in a VAP patient with culture identified methicillin resistance *Staphylococcus aureus* (MRSA). Coverage from 109 reads from Patient 2, time point 1 is shown for each base position of the 2.2 kilobase *mecA* gene.
(PDF)

S1 File Effect of quality control on Centrifuge’s taxonomic assignment and relative abundance estimates. (DOCX)

S2 File Effect of host sequence removal on taxonomic assignment in febrile neutropenia samples. (DOCX)
