# Supplementary figures and images for: Identification and quantitation of clinically relevant microbes in patient samples: Comparison of three k-mer based classifiers for speed, accuracy, and sensitivity
Source: PLoS Comput Biol. 2019 Nov 22;15(11):e1006863. doi: 10.1371/journal.pcbi.1006863 (PMC6897419; doi:10.1371/journal.pcbi.1006863)

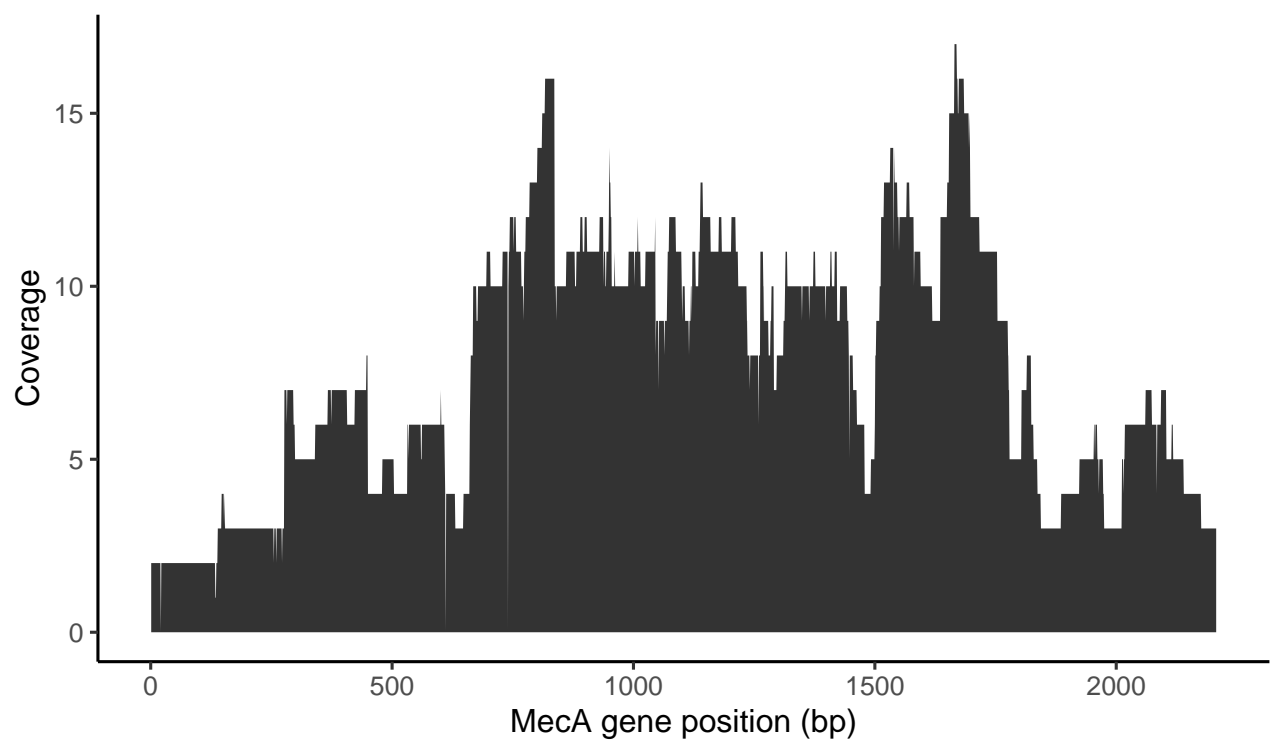

Supplement: S1 Fig — Coverage from 109 reads from Patient 2, time point 1 is shown for each base position of the 2.2 kilobase mecA gene. (PDF) [file pcbi.1006863.s002.pdf]
