# Supplementary material for: Identification and quantitation of clinically relevant microbes in patient samples: Comparison of three k-mer based classifiers for speed, accuracy, and sensitivity
Source: PLoS Comput Biol. 2019 Nov 22;15(11):e1006863. doi: 10.1371/journal.pcbi.1006863 (PMC6897419; doi:10.1371/journal.pcbi.1006863)
Supplement: S1 File — (DOCX) [file pcbi.1006863.s003.docx]

**S1 File**

**Effect of read quality control on Centrifuge’s taxonomic assignment and relative abundance estimates.**

**Staggered mock bacterial community reads.** Sequencing reads are typically subjected to a series of quality control steps including trimming low-quality bases from reads, removing short reads, deduplication, and trimming ends with unbalanced nucleotide composition before downstream applications (e.g., variant calling, or sequence assembly). When quality control steps were performed before Centrifuge analyses in Figs 1 and 2, they accounted for approximately half the compute time required to achieve results (data not shown). The fact that quality controls steps accounted for so much of the compute time, led to the question of what effect quality control had on the taxonomic classifications and relative abundance estimates made by Centrifuge. To answer this question, the staggered mock bacterial community sequence reads were classified by Centrifuge with and without quality control. Results showed only one difference in taxonomic classification: a false positive (*Bacillus thuringiensis*) was identified with a relative abundance of 2.9% without quality control (Fig A). Linear regression of the measured versus expected relative abundances showed that the R^2^ with and without quality control was 0.97, further demonstrating how little effect there was on the Centrifuge results. Interestingly, the abundance estimates for the no-QC data were closer to expected than with quality-controlled data (Fig A)


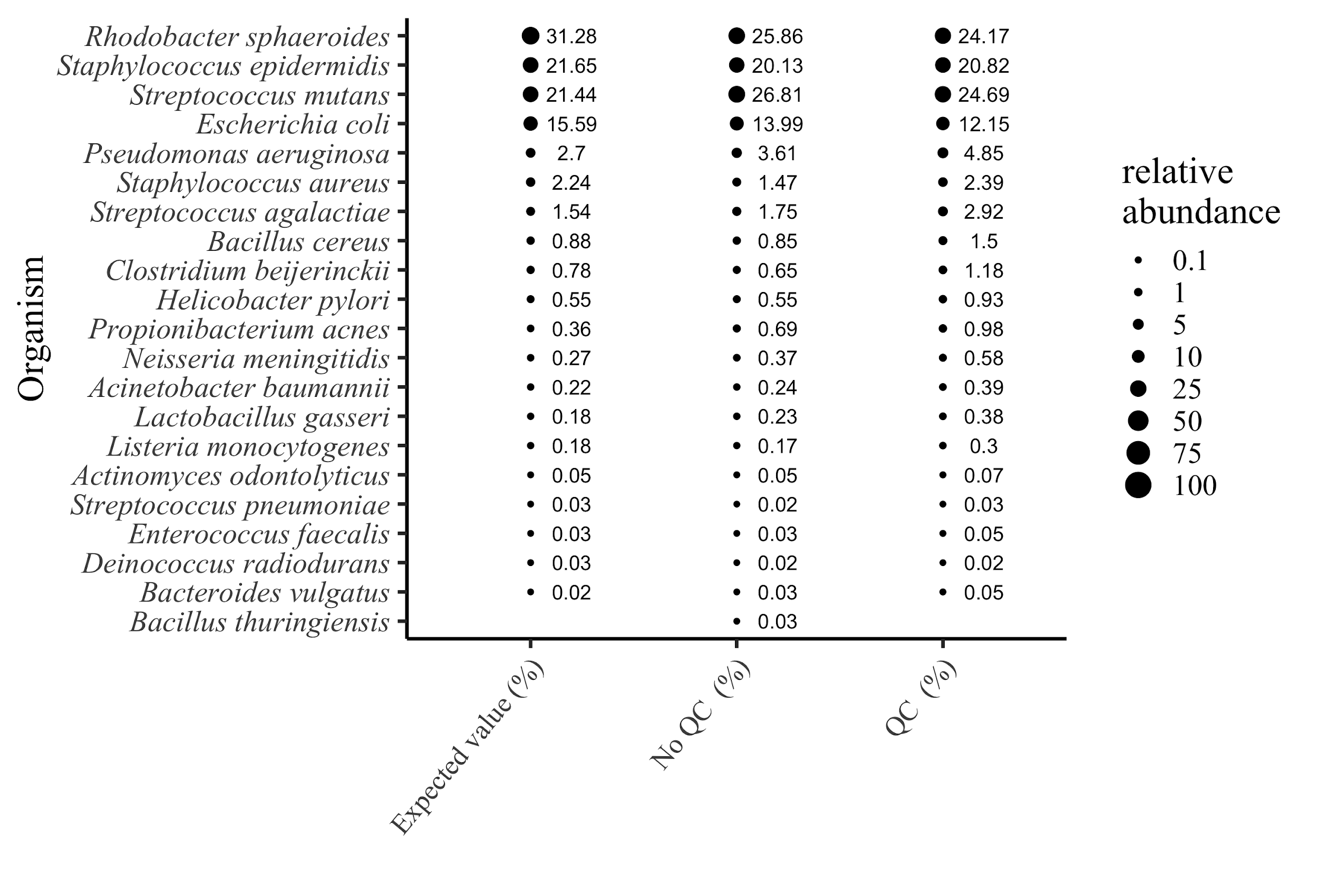


**Fig A.** **Effect of quality control of reads prior to analysis with Centrifuge on the staggered mock community taxonomic assignments and relative abundance estimates.** Identity and expected relative abundance with and without quality control are shown alongside expected results. Organisms are ranked by their relative abundance which is indicated by the size of the circle with actual percent values shown to the right. The single false positive (*Bacillus thuringiensis*) identified when no quality control was performed is shown at bottom.

**Quality control of febrile neutropenia reads**. Febrile neutropenia datasets were subjected to Centrifuge classification with and without prior quality control. Abundance estimates were generally the same, however, relative abundance for the Torque teno virus was 58% without QC and 50% with QC (Fig B). Despite the similar relative abundance estimates between no quality control and quality control, the sum of all bacterial and viral reads classified pre- and post-quality control showed a net read reduction skewed toward removing viral sequences (Table A).


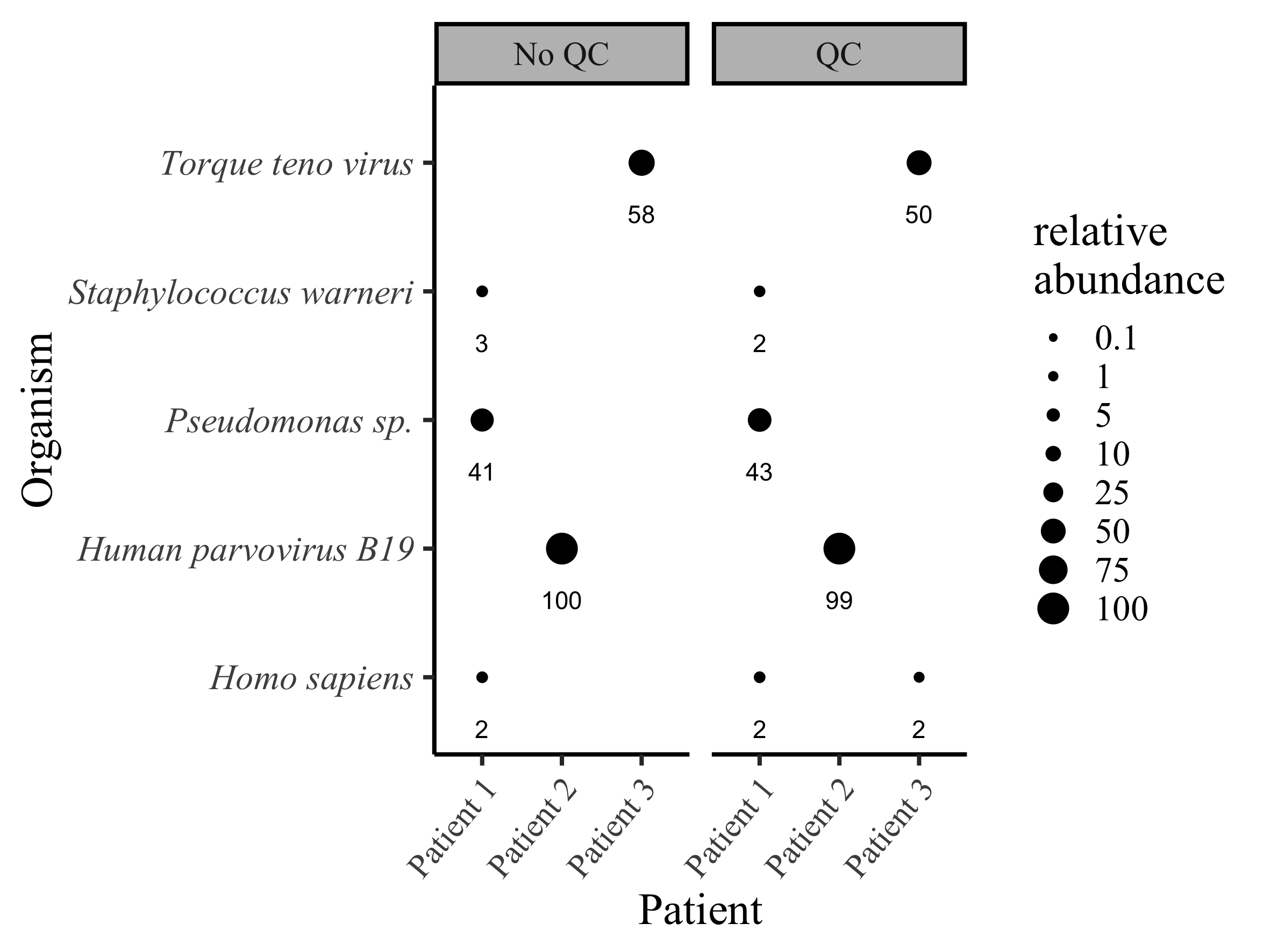


**Fig B. Taxonomic classification and relative abundance estimates by Centrifuge for febrile neutropenia samples with and without quality control of the reads prior to analysis.** Identity and estimated relative abundance with and without quality control are shown. The relative abundance of organisms identified is represented by circle size with actual values displayed below; values that are zero have no circle.

**Table A. Reduction of reads classified as bacterial and viral by quality control.**

| Classification | Net read reduction (%) | Mean read reduction |
| --- | --- | --- |
| Bacteria | 6.71 | 8,252 |
| Virus | 47.97 | 115,327 |
