# Supplementary material for: Identification and quantitation of clinically relevant microbes in patient samples: Comparison of three k-mer based classifiers for speed, accuracy, and sensitivity
Source: PLoS Comput Biol. 2019 Nov 22;15(11):e1006863. doi: 10.1371/journal.pcbi.1006863 (PMC6897419; doi:10.1371/journal.pcbi.1006863)
Supplement: S2 File — (DOCX) [file pcbi.1006863.s004.docx]

**S2 File**

**Effect of host sequence removal on taxonomic assignment in febrile neutropenia samples.**

Host DNA contamination can contribute to a significant proportion, or even the vast majority, of reads in metagenomic datasets, and is often removed by mapping reads to the host genome. In performing taxonomic classification of reads, Centrifuge determines whether reads are of human origin (or other hosts), thus calling into question the necessity of aligning reads to the host genome in order to remove them prior to analysis. Having established that a significant proportion of the reads in the datasets were of host origin (Table 3), we compared three approaches for removing host reads in the febrile neutropenia patient data: (1) alignment to the human genome and removal of aligned reads from the dataset, (2) removing the human sequence from the reference database, and (3) using the "exclude TaxID" function in Centrifuge to exclude reads from classification whose best match was to the human genome.

Overall, removal of host reads be any method led to higher relative abundance estimates for the presumed pathogens *Pseudomonas sp.* and torque teno virus (Fig A, compare to Fig 6), but caused additional likely spurious organisms to pass filtering including endogenous human retrovirus K113, *Staphylococcus warneri*, *Staphylococcus haemolyticus* and *micrococcus luteus*.

**
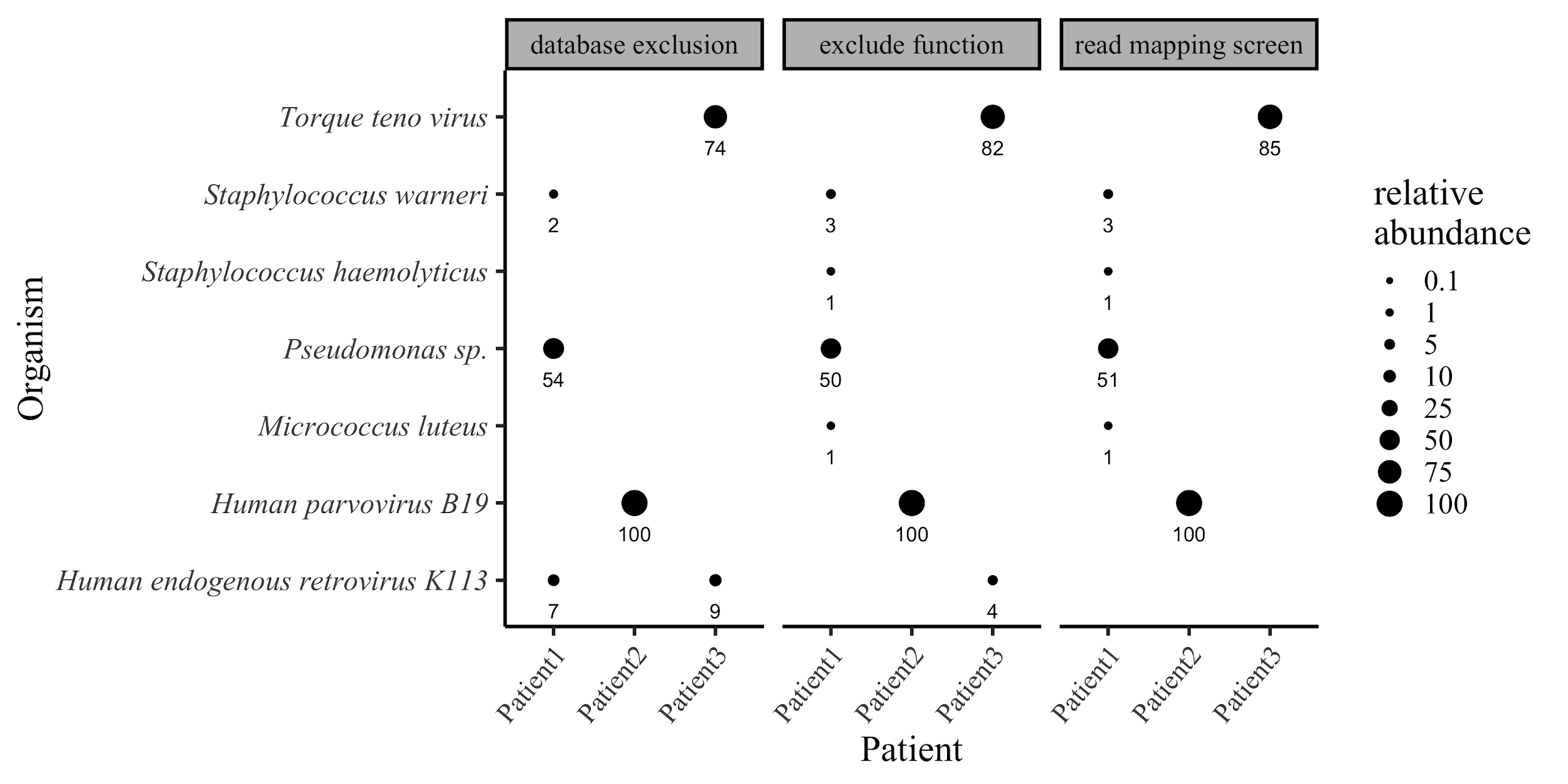
**

**Fig A. Effect of three methods of removing host (human) reads from febrile neutropenia patient datasets.** Circle size represents relative abundance calculated by Centrifuge with actual values shown below. Database exclusion, removal of the human genome from the reference database; exclude function, excluding the human TaxID in Centrifuge, read mapping screen, aligning against the human genome and removal of aligned reads before Centrifuge.
